# Many obesity-associated SNPs strongly associate with DNA methylation changes at proximal promoters and enhancers

**DOI:** 10.1186/s13073-015-0225-4

**Published:** 2015-10-08

**Authors:** Sarah Voisin, Markus Sällman Almén, Galina Y. Zheleznyakova, Lina Lundberg, Sanaz Zarei, Sandra Castillo, Fia Ence Eriksson, Emil K. Nilsson, Matthias Blüher, Yvonne Böttcher, Peter Kovacs, Janis Klovins, Mathias Rask-Andersen, Helgi B. Schiöth

**Affiliations:** Department of Neuroscience, Functional Pharmacology, Uppsala University, Uppsala, Sweden; Department of Medical Biochemistry and Microbiology, Uppsala University, SE-751 23 Uppsala, Sweden; Medical Faculty, IFB Adiposity Diseases, University of Leipzig, Liebigstrasse 21, 04103 Leipzig, Germany; Latvian Biomedical Research and Study Center, Ratsupites 1, Riga, LV-1067 Latvia

## Abstract

**Background:**

The mechanisms by which genetic variants, such as single nucleotide polymorphisms (SNPs), identified in genome-wide association studies act to influence body mass remain unknown for most of these SNPs, which continue to puzzle the scientific community. Recent evidence points to the epigenetic and chromatin states of the genome as having important roles.

**Methods:**

We genotyped 355 healthy young individuals for 52 known obesity-associated SNPs and obtained DNA methylation levels in their blood using the Illumina 450 K BeadChip. Associations between alleles and methylation at proximal cytosine residues were tested using a linear model adjusted for age, sex, weight category, and a proxy for blood cell type counts. For replication in other tissues, we used two open-access datasets (skin fibroblasts, *n* = 62; four brain regions, *n* = 121–133) and an additional dataset in subcutaneous and visceral fat (*n* = 149).

**Results:**

We found that alleles at 28 of these obesity-associated SNPs associate with methylation levels at 107 proximal CpG sites. Out of 107 CpG sites, 38 are located in gene promoters, including genes strongly implicated in obesity (*MIR148A*, *BDNF*, *PTPMT1*, *NR1H3*, *MGAT1*, *SCGB3A1*, *HOXC12*, *PMAIP1*, *PSIP1*, *RPS10-NUDT3*, *RPS10*, *SKOR1*, *MAP2K5*, *SIX5*, *AGRN*, *IMMP1L*, *ELP4*, *ITIH4*, *SEMA3G*, *POMC*, *ADCY3*, *SSPN*, *LGR4*, *TUFM*, *MIR4721*, *SULT1A1*, *SULT1A2*, *APOBR*, *CLN3*, *SPNS1*, *SH2B1*, *ATXN2L*, and *IL27*). Interestingly, the associated SNPs are in known eQTLs for some of these genes. We also found that the 107 CpGs are enriched in enhancers in peripheral blood mononuclear cells. Finally, our results indicate that some of these associations are not blood-specific as we successfully replicated four associations in skin fibroblasts.

**Conclusions:**

Our results strongly suggest that many obesity-associated SNPs are associated with proximal gene regulation, which was reflected by association of obesity risk allele genotypes with differential DNA methylation. This study highlights the importance of DNA methylation and other chromatin marks as a way to understand the molecular basis of genetic variants associated with human diseases and traits.

**Electronic supplementary material:**

The online version of this article (doi:10.1186/s13073-015-0225-4) contains supplementary material, which is available to authorized users.

## Background

Genome-wide association studies (GWASs) have identified a plethora of common genetic variants that are associated with obesity-associated traits (e.g., body mass index (BMI) [1–11], fat mass [12, 13], low lean body mass [14], blood lipid levels [15], waist circumference [13, 16], BMI-adjusted waist-to-hip ratio [17, 18]). Some of these single nucleotide polymorphisms (SNPs) are located near genes whose role in obesity is well established, such as *MC4R* [19]. However, most of these SNPs are located near genes whose role in obesity is still unclear, and the mechanisms through which they act remain unknown. Part of this lack of understanding may be due to a focus on the genes in closest proximity to these SNPs. Actually, these SNPs may regulate genes that are located quite far away, as recently demonstrated for genetic variants within *FTO*. In human brains, obesity-associated SNPs in *FTO* were found to be associated with expression of *IRX3*, a gene located more than half a million base pairs downstream of the body mass-associated genetic locus [20]. Another instance is the rs4537545 SNP previously associated with coronary heart disease [21] and located within *IL6R*: this SNP was recently found to be associated with blood mRNA levels of *ATP8B2*, a gene located 115 kb away [22]. Thus, obesity-associated SNPs might act through long-range interactions (for example, by disrupting enhancers) and potentially through epigenetic mechanisms.

The epigenome represents the pattern of chemical and structural modifications to DNA that are heritable through mitosis and/or meiosis, but that do not entail changes in DNA sequence. Epigenetic mechanisms encompass DNA methylation, histone modifications, and non-coding RNAs, and have the potential to modify gene expression. Recent attention has been drawn to the possible role of epigenetics in the pathogenesis of obesity [23, 24]. Moreover, while the epigenome is known to be modulated by the environment, this modulation can also be affected by genetic variants. Studies in brain [25–28], adipose tissue [29, 30], blood [26, 31, 32], lung [33], fibroblasts [34, 35], T cells [35], leukocytes [36], and lymphoblastoid cells [35, 37] have shown that the genome contains quantitative trait loci (QTLs) for DNA methylation, also called methylation QTLs (meQTLs). DNA methylation levels correlate with the presence of specific alleles at nearby SNPs, and meQTLs tend to locate outside of promoters, especially in intergenic regions. In a study conducted in adipose tissue [29], meQTLs overlapping metabolic disease loci were enriched in histone marks predictive of genetic enhancers. Interestingly, top associations from a GWAS of bipolar disorder were enriched in meQTLs [38], suggesting that this could be a powerful approach to better understand the molecular basis of candidate SNPs from GWASs.

In the present study, we tested associations between 52 SNPs that were previously identified in GWASs or meta-analyses to be associated with obesity traits, and proximal DNA methylation in whole blood of 355 healthy young individuals. We then tested the tissue specificity of the majority of these associations in four brain regions (*n* = 121–133), visceral adipose tissue (VAT; *n* = 149), subcutaneous adipose tissue (SAT; *n* = 149) and fibroblasts (*n* = 62). Finally, the genomic context of associated CpG sites was explored, using chromatin segmentation on publicly available histone marks from 11 tissues and long-range interactions from five cell lines.

## Methods

### Discovery study group

#### Ethics, consent and permissions

The discovery study group comprised two sub-groups of healthy young Caucasians from two different age ranges (Table 1). All participants and their guardians gave informed written consent and the study was approved by the local ethics committee in Uppsala, EPN, diary number 2011/446; this study was conducted in accordance with the principles of the Declaration of Helsinki. The first sub-group comprised 130 individuals aged 14–16 years who were recruited by visiting schools in Uppsala county and by post. Two 6-ml blood samples were drawn for genotyping and DNA methylation measurement, at any time during the day. The other sub-group comprised 225 individuals of white European descent aged 18–34 years, also recruited in Uppsala. Subjects were fasting (at least 10 h) when blood samples were taken for genotyping and DNA methylation measurement. For individuals aged under 18 years, we used Cole et al.’s definition to determine weight category [39]. For individuals aged 18 years and older, the following cutoffs were used: lean, BMI < 25; overweight, 25 ≤ BMI < 30; obese, BMI ≥ 30. We chose to use weight category instead of BMI since our cohort includes individuals aged under 18 years whose BMI scales differ from the BMI scales of individuals aged over 18 years.

**Table 1:** Description of the discovery samples

	Sub-group 1	Sub-group 2	Total
n	130	225	355
n_males_	37 (29 %)	177 (79 %)	214 (60 %)
Age (years)^a^	15.3 ± 0.64	23.6 ± 3.3	20.6 ± 1.2
Weight (kg)^a^	72.9 ± 11.4	76.6 ± 12.4	71.6 ± 13.7
Height (m)^a^	1.70 ± 0.081	1.79 ± 0.078	1.76 ± 0.092
Weight category^b^	77 % lean, 18 % overweight, 5 % obese	74 % lean, 21 % overweight, 5 % obese	76 % lean, 20 % overweight, 4 % obese

^a^Mean ± standard deviation. ^b^For individuals aged under 18 years, we used Cole et al.’s definition to determine weight category [39]. For individuals aged 18 years and older, the following cutoffs were used: lean, BMI < 25; overweight, 25 ≤ BMI < 30; obese, BMI ≥ 30

#### Genotyping

We selected 52 SNPs that have been associated by GWASs or meta-analyses of GWASs with obesity-associated traits (BMI [1–10], BMI-adjusted waist-to-hip ratio [18], fat mass [12], low lean body mass [14], blood lipid levels [15] and waist circumference [16]) and the discovery study group was genotyped for these SNPs (Additional file 1). Genotyping of the 52 SNPs was carried out at the SNP technology platform at Uppsala University [40] using an Illumina Golden Gate Assay (Illumina Inc., San Diego, CA, USA). There were missing genotypes for 8 of the 52 tested SNPs, ranging from one individual to 52 individuals with missing genotypes (Additional file 1). Individuals with missing genotypes were removed from the analysis.

#### DNA methylation profiling

The genome-wide Illumina Infinium HumanMethylation450 BeadChip (Illumina), which allows interrogation of 485,512 CpG dinucleotides covering 25,953 genes, was applied to determine the methylation profile of genomic DNA isolated and purified from the peripheral whole blood. This chip has been shown to give a reliable and reproducible estimation of the methylation profile on a genomic scale [15]. First, bisulfite conversion of genomic DNA was performed using the EZ DNA Methylation-Gold™ Kit (Zymo Research) according to the manufacturer’s protocol. Briefly, 500 ng of DNA was sodium bisulfite-treated, denatured at 98 °C for 10 min, and bisulfite converted at 64 °C for 2.5 h. After conversion, samples were desulfonated and purified using column preparation. Approximately 200 ng of bisulfate-converted DNA was processed according to the Illumina Infinium Methylation Assay protocol. This assay is based on the conversion of unmethylated cytosine (C) nucleotides into uracil/thymine (T) nucleotides by the bisulfite treatment. The DNA was whole-genome amplified, enzymatically fragmented, precipitated, resuspended, and hybridized overnight at 48 °C to locus-specific oligonucleotide primers on the BeadChip. After hybridization, the C or T nucleotides were detected by single-base primer extension. The fluorescence signals corresponding to the C or T nucleotides were measured from the BeadChips using the Illumina iScan scanner. Phenotypes, genotypes, raw data, and processed DNA methylation data are available through the Gene Expression Omnibus (GEO) database [41] with accession number [GEO:GSE73103].

#### DNA methylation processing

All downstream data processing and statistical analyses were performed with the statistical software R [42] together with the *minfi* [43], *ChAMP* [44], *sva* [45], and *MethylAid* [46] packages of the Bioconductor project.

#### Background correction and adjustment of type I and type II probes

Fluorescence data were preprocessed using the GenomeStudio 2009.2 (Illumina) software. First, we background corrected the data using NOOB [47]. In the Illumina Infinium HumanMethylation450 BeadChip array, the probes come in two different designs, characterized by widely different DNA methylation distributions and dynamic range, which may bias downstream analyses. Therefore, we applied the BMIQ algorithm to adjust for the two different probe designs [48].

#### Removal of batch effects

The plates on which samples are run introduce a known batch effect that is important to correct for. We used the ComBat function to adjust directly for this batch effect [45].

#### Principal component analysis

We performed a principal component analysis (PCA) using the PCA function of the *FactoMineR* package [49], first calculating the covariance matrix between all samples using only the most variable autosomal CpG sites, measured in terms of their 95 % reference range: the range of methylation values observed in the central 95 % of the samples or, more precisely, the difference between the 97.5 and 2.5 % percentiles. Using a 95 % reference range of at least 0.20, 103,408 CpG sites were used in the covariance matrix calculation. Together, the two first principal components explain over 39 % of the total variance. Each subsequent vector does not add substantially to the variance explained: 285 vectors would be necessary to explain 95 % of the total variance.

#### Sample exclusion

We excluded from association analyses: (1) samples that were outliers in any one of the quality control plots generated by MethylAid [46] (rotated M versus U plot, overall sample-dependent control plot, bisulfite conversion control plot, overall sample-independent control plot and detection *p* value plot) using the default thresholds (0 samples); (2) samples that were outliers with respect to any one of the first eight principal components (corresponding to the approximate location of the elbow of the eigenvalue scree plot; six samples). After exclusion of samples, we were left with 349 samples: 128 from the first sub-group (29 % males; mean age ± standard deviation 15.3 ± 0.64 years) and 221 from the second sub-group (78 % males; mean age ± standard deviation 23.6 ± 3.3 years).

#### Probe exclusion

We removed probes with missing β values, probes having less than 75 % of samples with detection *p* value < 0.01, and probes located on the sex chromosomes. Using the annotation generated by Chen et al. [50], we also removed cross-reactive probes and probes containing SNPs with minor allele frequency > 1 % in European populations. In total, 397,615 probes were included in the analysis.

#### Choice of investigated CpGs

We selected the probes within 500 kb of each SNP. A total of 8485 probes were analyzed, with an average of 163 CpGs per SNP (Additional file 1).

#### Cell-type proportions

Because differences in cell-type proportions between DNA samples can confound association results [51], we adjusted our analyses using a surrogate for cell-type proportions derived from 43 differentially methylated CpG sites present on the HumanMethylation450 array that have the ability to discriminate between blood cell types [52]. As a surrogate for cell-type proportions, and to reduce the number of variables, we used the first two principal components associated with these 43 sites that together explain over 70 % of the total variance in methylation at these 43 CpG sites.

To verify that the first two principal components that we derived from the list of 43 differentially methylated CpG sites [52] can indeed serve as a surrogate for blood cell proportions, we tested for associations between the principal components and the methylation levels at all of our sites, adjusting our analyses for sex, age, weight category, and batch. We selected the top 10 % of the sites that showed the strongest associations (49,035 sites, all associated at levels *p* < 10^−8^) and extracted these sites in data sets of purified human leukocyte subtypes [53] [GEO:GSE39981]; 2564 sites were overlapping. A dendrogram representation of our top sites in this data set [53] reveals clear clustering of samples according to cell type, indicating a good ability for principal components to discriminate between samples with different cell compositions (Additional file 2).

#### Validation of methylation with bisulfite sequencing

The methylation levels of two of the associated CpG sites (cg15576492 and cg2204028, at position chr1:1015257–1015540) were validated using bisulfite sequencing. The sequences including target CpG sites were obtained from the University of California, Santa Cruz (UCSC) Genome Browser database. The sequences (bisulfite-converted DNA template) for the primers were forward (biotin labeled)-5′-ATGGATGTTGGTGTGAGTATT-3′ and reverse 5′-CCCTCTACACATCTAAACCCT-3′. Bisulfite sequencing primers were designed with Methyl Primer Express® v.1.0 (Applied Biosystems) so that the amplicons covered target CpG sites. These regions were PCR amplified in duplicate from bisulfite-treated DNA. Similar efficiency in PCR amplification for unmethylated and methylated fragments was controlled for using Human Methylated & Non-methylated DNA Set (Zymo Research). PCR reactions were performed in a final volume of 25 μl and contained 2.5 μl of bisulfite-treated DNA (10–15 ng/μl), 0.05 μl of each primer (100 pmol/μl), 1 μl DMSO, 0.5 μl of SYBR Green I (1:50,000; Invitrogen, Sweden) in TE buffer (pH 7.8), 0.25 μl of 25 mM dNTP mix (Fermentas), 2.5 μl 10× buffer, 4 μl of 25 mM MgCl_2_, 1 U of Hot Start Taq DNA polymerase (Thermo Scientific). Cycling conditions were as follows: 10-min initial denaturation step at 95 °C, followed by 45 cycles of 95 °C for 20 s, 30 s at optimal annealing temperature of primers, 20–45 s at 72 °C, 5 min of final elongation at 72 °C. Fluorescence was measured after the elongation phase. Melting curve analysis consisted of 81 cycles of 10 s at 55 °C with increasing increments of 0.5 °C per cycle. Bio-Rad iQ5 version 2.0 software (Bio-Rad Laboratories) was used to process real-time PCR data.

Amplicons were purified using GeneJET PCR Purification Kit (Thermo Scientific).

DNA sequencing was performed using BigDye® Terminator v.3.1 Cycle Sequencing Kit (Applied Biosystems) on an ABI3730XL DNA Analyzer (Applied Biosystems) at Uppsala Genome Center. Cycle sequencing was as follows: 30 s initial denaturation step at 94 °C, followed by 35 cycles of 94 °C for 25 s, 50 °C for 15 s, 60 °C for 120 s. Each sample was sequenced twice and the two methylation levels were averaged. Amplification primers were used for sequencing. All samples were analyzed in duplicates on different plates and the mean methylation levels in percentage per sample were used for further analyses. Methylation levels of CpG sites for all amplicons were quantified using Epigenetic Sequencing Methylation analysis software [54]. The software was repeatedly used to determine the methylation profile of several genes [55, 56]. The software algorithm analyzes the methylation percentage of each CpG site in an amplicon without cloning stage.

### Replication study groups

#### VAT and SAT

VAT and SAT samples were used to test specifically the association between alleles at rs1011731 and methylation at cg13446689. Paired samples of VAT and SAT from 149 Caucasian subjects (35 % male) who underwent open abdominal surgery were included in the study. This subset is part of a study group that had already been genotyped for rs1011731, described in detail elsewhere [57]. Thirty-two individuals were lean (aged 63 ± 11 years, BMI 22.1 ± 2.5 kg/m^2^), 22 were overweight (67 ± 12 years, BMI 27.1 ± 1.4 kg/m^2^) and 94 were obese (age 47 ± 13 years, BMI 48.1 ± 9.7 kg/m^2^); BMI was missing for one individual and 46 subjects had diabetes type 2. Patients with severe conditions, including generalized inflammation or end-stage malignant diseases, were excluded from the study. Samples of VAT and SAT were immediately frozen in liquid nitrogen after explantation. The study was approved by the ethics committee of the University of Leipzig and all subjects gave written informed consent.

Genomic DNA was extracted from frozen adipose tissue samples using GenElute™ Mammalian Genomic DNA Miniprep Kit (SIGMA-ALDRICH, USA). All samples were bisulfite converted using Qiagen Epitect Bisulfite Kit (Qiagen, Hilden, Germany) according to the manufacturer’s protocol and applied to whole bisulfitome amplification (EpiTect Whole Bisulfitome Kit, Qiagen, Hilden, Germany). Finally, all samples were purified using GenElute PCR Clean-up Kit (Sigma-Aldrich, USA). Methylation levels of cg13446689 were determined using a custom designed PyroMark CpG assay (Qiagen, Hilden, Germany). The sequences (bisulfite-converted DNA template) for the primers were forward (biotin labeled)-5′-AAGTGATGGGAGTTGTTGG-3′ and reverse 5′- ACCCCAAAACAATTCAAACAAACCATA-′3. Using the sequencing primer 5′-ACAATTCAAACAAACCATACTTA-3′ the following sequence was analyzed (5′- CACAAC[R]ACTAACTAATCTATAC[R]ACCTCAAACCAAAAACAACAACCAACAACTCC-3′). The pyrosequencing was run on a PyroMark Q24 (Qiagen, Hilden, Germany). All samples were analyzed in duplicates on different plates and the mean methylation levels in percentage per sample were used for further analyses. Water was used as a non-template control using the same PCR conditions.

#### Fibroblasts

Methylation, SNP genotyping, and gene expression data from primary skin fibroblasts from Caucasian individuals (*n* = 62) [34] were obtained from GEO (accession number [GEO:GSE53261]).

#### Brain regions (cerebellum, frontal cortex, caudal pons, and temporal cortex)

SNP genotyping data from four different brain regions (*n* = 121–133) [58] were obtained from dbGAP (accession number phs000249.v1.p1). All individuals were of Caucasian descent, but two individuals from the cerebellum study samples were of African and Asian descent, respectively. We removed these two individuals from our analysis. Methylation data were obtained from GEO (accession number [GEO:GSE15745]).

### Annotation

#### Genes

The genomic positions of RefSeq genes were downloaded from the UCSC genome browser, and the location of each CpG site was determined as promoter (within 1500 bp of the transcription start site (TSS)), gene body, intergenic, or ambiguous (overlapping a promoter and a gene body).

#### Linkage disequilibrium

Linkage disequilibrium (LD) data were obtained from SNAP Proxy, using CEU as the “population panel” and the 1000 Genomes Pilot 1 as “SNP dataset” [59].

#### Chromatin states

ChromHMM [60] was applied for seven publicly available histone modifications (H3K4me1, H3K4me3, H3K9ac, H3K9me3, H3K27ac, H3K27me3, and H3K36me3) from 11 tissues: adipose nuclei (AN), pancreatic islets (PI), peripheral blood mononuclear primary cells (PBMC), skeletal muscle (SM), liver, brain angular gyrus (BrainAG), brain anterior caudate (BrainAC), brain cingulate gyrus (BrainCG), brain hippocampus (BrainHIPPO), brain inferior temporal lobe (BrainITL), and brain substantia nigra (BrainSN). Data were downloaded from NIH Roadmap Epigenomics Project Data Listings. An 18-state model was learned from all binarized data and was used to produce segmentations based on the most likely state assignment of the model. Then, each state was assigned to one of the following seven categories: enhancer, active TSS/poised TSS/flanking TSS, active transcription, quiescent, heterochromatin, Polycomb-repressed, ZNF genes/repeats.

#### Ubiquitous, tissue-specific, and cell-specific *in vivo* transcribed enhancers

Ubiquitous, tissue-specific (adipose tissue, blood, brain, liver, pancreas, and skeletal muscle) and cell type-specific (preadipocytes, fat cells, hepatocytes, and skeletal muscle cells) enhancers, as well as TSS–enhancer associations, as defined by CAGE tags in the FANTOM5 project, were downloaded from the Transcribed Enhancer Atlas website [61, 62].

#### Long-range interactions

We used publicly available chromatin interaction analysis by paired-end tag sequencing (ChIA-PET) libraries to map long-range interactions in five different cell lines, with three different transcription factors [63] (Additional file 3). Data were downloaded from the WashU Epigenome Browser.

#### Expression QTLs

We used the following publicly available expression QTL (eQTL) browsers to see whether any of the associated SNPs or SNPs in strong linkage with them (r^2^ > 0.8) were eQTLs for our genes of interest: the eQTL browser of the Genotype-Tissue Expression (GTEx) project [64], the eQTL Browser of the National Center for Biotechnology Information, the eQTL resources from the Gilad/Pritchard group [65], and the blood eQTL browser developed by Westra *et al.* [66].

### Statistics

For statistical analysis, we used the log_2_ ratio of the intensities of methylated probe and unmethylated probe, also called M value, which is more statistically valid for the differential analysis of methylation levels [67].

#### Linear model

We developed the following linear model for each CpG site k:

$$ {M}_k={a}_k+{b}_{kS}S+{b}_{kA}A+{b}_{kW}W+{b}_{kG}G+{b}_{kPC1}PC1+{b}_{kPC2}PC2+{\varepsilon}_k $$

where M_k_ is the M value of CpG site k, S is the dichotomized sex (female = 1 and male = 0), A is the age, W is the weight category (normal weight = 0, overweight = 1, obese = 2), G is the genotype at the investigated SNP (homozygotes for non-risk allele = 0, heterozygotes = 1, homozygotes for risk allele = 2), PC1 and PC2 are the first two principal components derived from the list of 43 differentially methylated CpG sites in blood cell types, and ε_k_ is the unexplained variability. We chose to use weight category instead of BMI since our study samples include individuals aged under 18 years whose BMI scales differ from the BMI scales of individuals aged over 18 years. Rare homozygous genotypes (count of less than 10) were combined with heterozygotes.

The coefficients *b*_*kx*_ summarize the association between methylation levels and the variables of interest. The *p* value for the SNP was determined using a likelihood ratio test, using the lrtest function of the *lmtest* package [68], and we report the effect size as the proportion R^2^ of the CpG methylation variance that is explained by the SNP, among the variance not already explained by the covariates. To control the proportion of false positives, q values were calculated using the qvalue function of the *qvalue* package [69]. A SNP was considered significant if its q value was < 0.05.

#### Enrichment of associated CpGs in genomic regions, in vivo transcribed enhancers, and chromatin states

To test whether associated CpGs were enriched or underrepresented in different genomic regions (promoter, gene body, etc.), chromatin states (enhancer, TSS, heterochromatin, etc.) and in vivo transcribed enhancers, we used Fisher’s exact test. To control the proportion of false positives, q values were calculated using the qvalue function of the *qvalue* package [69]. Significance was considered at a q value < 0.05.

#### Number of long-range interactions

The distributions of the numbers of long-range interactions per CpG were skewed. Thus, to see whether associated CpGs had a higher or lower number of long-range interactions, we used Mann–Whitney U-test.

#### Power calculations

We used the pwr.f2.test function of the *pwr* package in R to determine the statistical power in the replication datasets (fibroblasts, brain and SAT/VAT).

## Results

### Obesity-associated SNPs associate with methylation at proximal CpGs in whole blood samples from healthy individuals

**Fig. 1:**
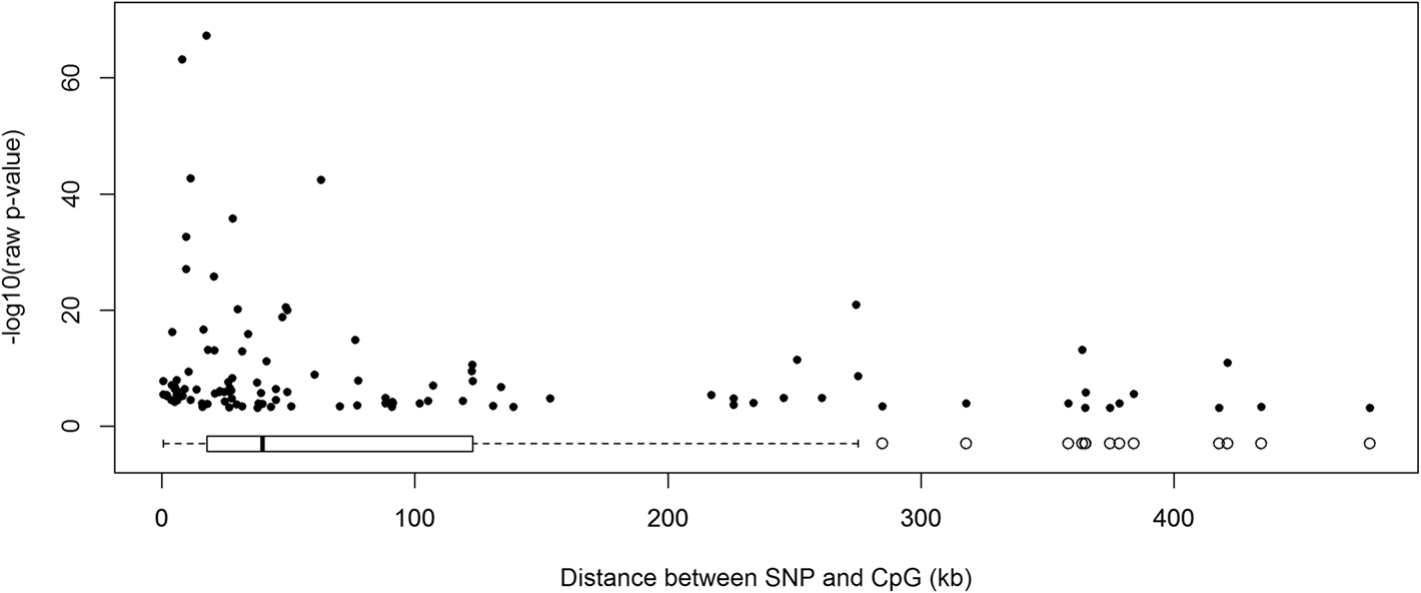
Raw *p* value as a function of distance between SNP and CpG. Each point represents an associated SNP–CpG pair (107 pairs). Most associated SNP–CpG pairs are close to each other, as illustrated by the box plot of the distance between SNP and CpG (bottom of the plot). The closer the associated SNP–CpG pairs are, the lower the *p* value

**Fig. 2:**
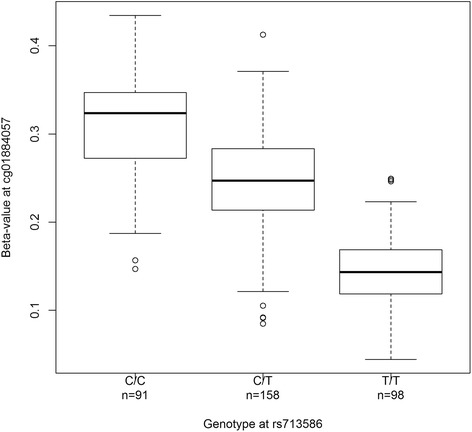
Associations between genotypes at rs713586 and methylation at cg01884057. Distribution of methylation levels at cg01884057 is displayed for individuals carrying zero (C/C), one (T/C), or two (T/T) risk alleles at rs713586

We tested associations between 52 obesity-associated SNPs and M values of all CpG sites 500 kb upstream and 500 kb downstream of each SNP in the blood of 355 individuals (Table 1), using a linear regression model adjusted for age, sex, blood cell type surrogate, and weight category (i.e., lean, overweight, or obese) instead of BMI since our study samples include individuals aged under 18 years whose BMI scales differ from the BMI scales of individuals aged over 18 years. In total, 8485 probes were tested, with an average of 163 probes per SNP (Additional file 1). Methylation levels at 107 CpGs associated with genotypes at 28 SNPs (likelihood ratio test, q value < 0.05; Additional file 4) and most of the associations were between SNPs and CpGs that are close to each other (50 % of the associations are between SNPs and CpGs that are within 40 kb of each other; Fig. 1). Also, the closer the SNP and CpG are, the stronger the statistical significance is (Fig. 1). One example of these SNP–CpG associations is depicted in Fig. 2. The rs713586 SNP explains 53.8 % of the total variance in methylation at cg01884057, with carriers of the risk allele (*C*) at rs713586 having higher methylation.

The two sub-groups that were pooled for the discovery analysis were of two different age ranges (see “Methods”), but they did not significantly differ in terms of global DNA methylation patterns, as shown by PCA (Additional file 5). To make sure that the two sub-groups were comparable and could effectively be combined for the discovery analysis, we tested the significance of the 107 CpGs separately in each. SNP effects were in the same directions for all 107 CpGs in the two separate sub-groups; 105 of the 107 CpGs were significant (raw *p* value < 0.05) in the first, while 86 of the 107 CpGs were significant (raw *p* value < 0.05) in the second. This suggests that our results are not driven by a specificity of one of the two sub-groups and that it was reasonable to pool them for the discovery analysis.

### Genomic context of CpGs associated with obesity-associated SNPs

To understand the functional significance of the CpGs associated with alleles at obesity-associated SNPs, we analyzed their genomic location in relation to genes, chromatin states in 11 tissues, ubiquitous, tissue-specific, or cell-specific in vivo transcribed enhancers, and long-range interactions in five cell lines.

#### CpGs associated with obesity-associated SNPs are depleted in promoters and enriched in intergenic regions

**Fig. 3:**
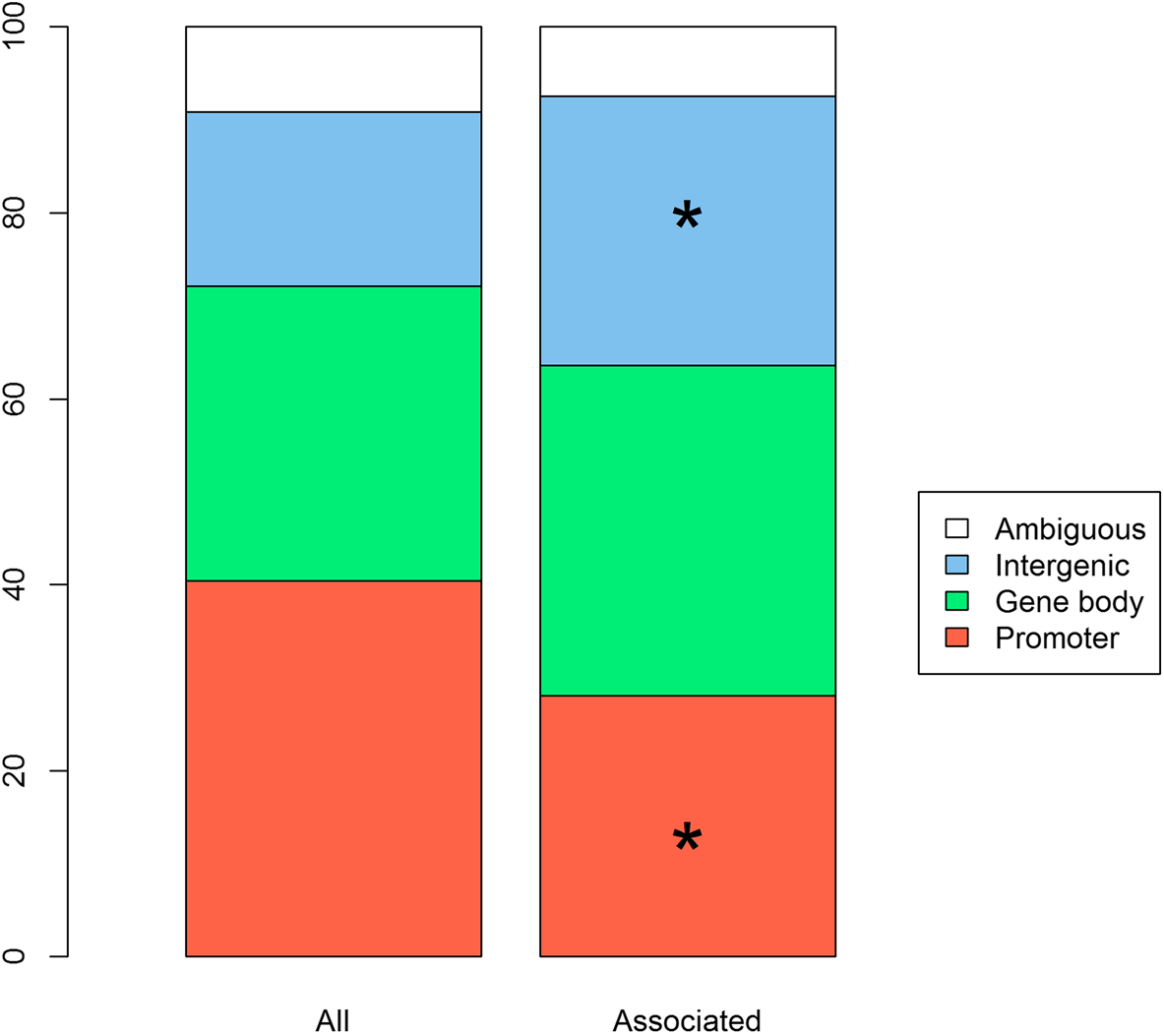
Distribution of associated versus all tested CpGs in promoters, gene bodies, and intergenic regions. A CpG was classified as “promoter” if located within 1500 bp of the TSS of a gene, and as “ambiguous” if it was both in a promoter and within a gene body. Associated CpGs (*top*) were underrepresented in promoters, and overrepresented in intergenic regions (Fisher’s exact test). *q value < 0.05

Thirty-eight of the associated CpGs were located in gene promoters (*MIR148A*, *BDNF*, *PTPMT1*, *NR1H3*, *MGAT1*, *SCGB3A1*, *HOXC12*, *PMAIP1*, *PSIP1*, *RPS10-NUDT3*, *RPS10*, *SKOR1*, *MAP2K5*, *SIX5*, *AGRN*, *IMMP1L*, *ELP4*, *ITIH4*, *SEMA3G*, *POMC*, *ADCY3*, *SSPN*, *LGR4*, *TUFM*, *MIR4721*, *SULT1A1*, *SULT1A2*, *APOBR*, *CLN3*, *SPNS1*, *SH2B1*, *ATXN2L*, and *IL27*), including eight also located in a gene body (Additional file 4). Thus, associated CpGs were underrepresented in promoters (28 % of CpGs, Fisher’s exact test *p* value = 0.0097). In contrast, 31 associated CpGs were located in intergenic regions, which is more than expected by chance (30 % of CpGs, Fisher’s exact test *p* value = 0.0087; Fig. 3). This is consistent with previous studies on meQTLs [27, 30].

#### CpGs associated with obesity-associated SNPs are enriched in enhancers in PBMCs

**Fig. 4:**
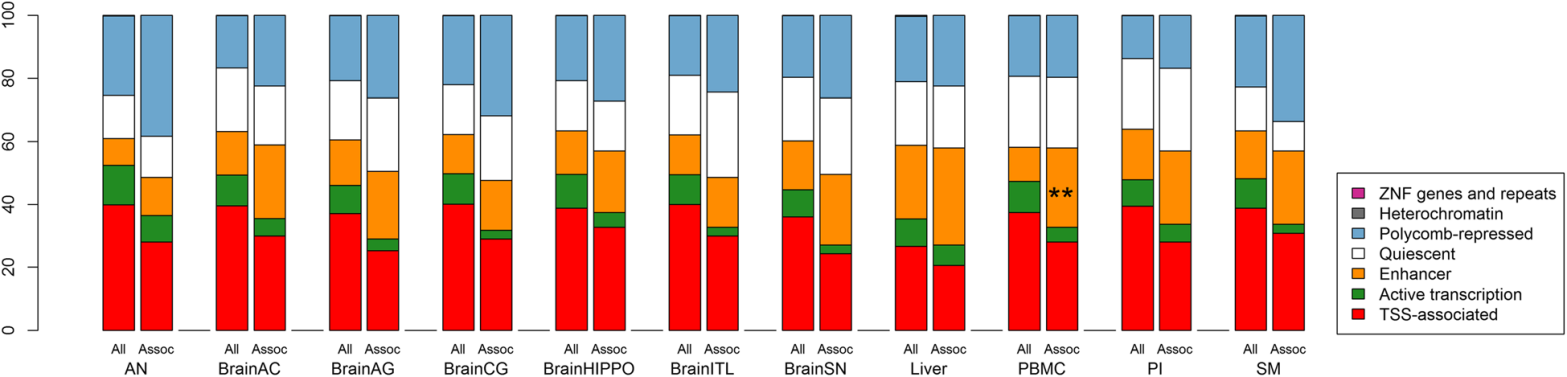
Distribution of associated versus all tested CpGs in seven chromatin states in 11 tissues. Associated CpGs were overrepresented in enhancers in PBMCs (Fisher’s exact test). **q value < 0.01

The activity of functional genomic elements is associated with the state of the chromatin at these sites, such as histone modification patterns and access of transcription factors to DNA. The recently developed chromHMM tool allows interpreting chromatin states in a particular tissue or cell type by integrating histone marks and transcription factor binding data [60]. Using seven publicly available histone marks in 11 tissues relevant in the pathogenesis of obesity (AN, six brain regions, liver, PBMCs, PIs, and SM), we interpreted the chromatin states of all regions containing the tested CpGs (Additional file 6). Consistent with the enrichment of associated CpGs in intergenic regions (Fig. 3), associated CpGs were enriched in enhancers in PBMCs (Fisher’s exact test, q value = 0.0019) (Fig. 4).

#### Only one CpG associated with obesity-associated SNPs is located in in vivo transcribed enhancers

The enrichment of associated CpGs in enhancers is a prediction by chromHMM that relies on histone marks, but we wanted to test whether associated CpGs were also found in active enhancers, as defined by cap-analysis of gene expression (CAGE) in the FANTOM5 project [61]. cg04588972, whose methylation was lower in carriers of the risk allele at rs1878047, was in a ubiquitous enhancer showing long-range interactions with the TSS of *KLK14*, *IGLON5*, *LRRC4B*, and *SYT3*. However, associated CpGs were not underrepresented in ubiquitous, tissue-specific, or cell-specific enhancers (Fisher’s exact tests, all *p* values > 0.05). FANTOM5 uses very stringent criteria to detect active enhancers using whole transcriptome sequencing [61], thus possibly explaining why none of the associated CpGs were in active enhancers as defined by CAGE in the FANTOM5 project. Indeed, chromHMM predicted 33–266 times more active enhancers from the FANTOM5 project depending on the tissue, and there was little overlap between the two.

#### CpGs associated with obesity-associated SNPs show long-range interactions with promoters and other genomic regions

**Fig. 5:**
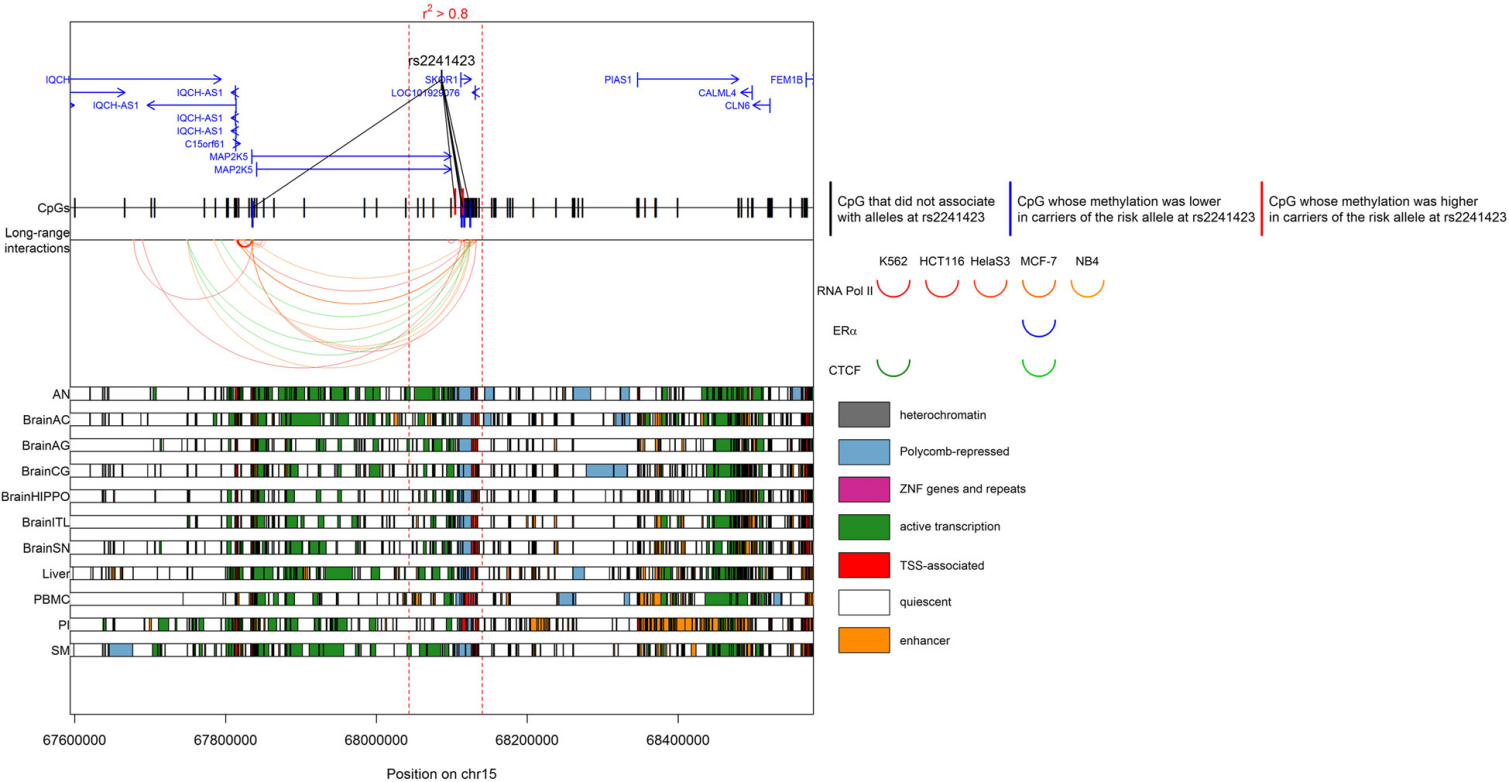
Genomic context of the CpGs associated with rs2444217. Genomic positions of RefSeq genes and rs2444217 are displayed in the *top panel*. Within the two *vertical red dotted lines*, the LD r^2^ > 0.8. The positions of the tested CpGs are displayed. Long-range interactions as defined by ChIA-PET libraries from five cell lines using chromatin immunoprecipitation with antibodies targeting three transcription factors (Additional file 5) are displayed as arcs. For clarity of visualization, we chose to display only the long-range interactions of genomic regions containing associated CpGs. Two interacting genomic regions are represented by an arc that links them, and the thickness of the arc line is proportional to the strength of this interaction. The color of the arc corresponds to the target transcription factor and the shade of the color corresponds to the cell line: *red* for RNA polymerase II, *blue* for ERα, and *green* for CTCF. In the *bottom panel*, chromatin states in 11 tissues are displayed. Chromatin states were obtained using chromHMM prediction using data on seven histone marks (see “Methods”). The color of each band corresponds to a particular state. *AN* adipose nuclei, *BrainAC* brain anterior caudate, *BrainAG* brain angular gyrus, *BrainCG* brain cingulate gyrus, *BrainHIPPO* brain hippocampus, *BrainITL* brain inferior temporal lobe, *BrainSN* brain substantia nigra, *PBMC* peripheral blood mononuclear primary cells, *PI* pancreatic islets, *SM* skeletal muscle

**Fig. 6:**
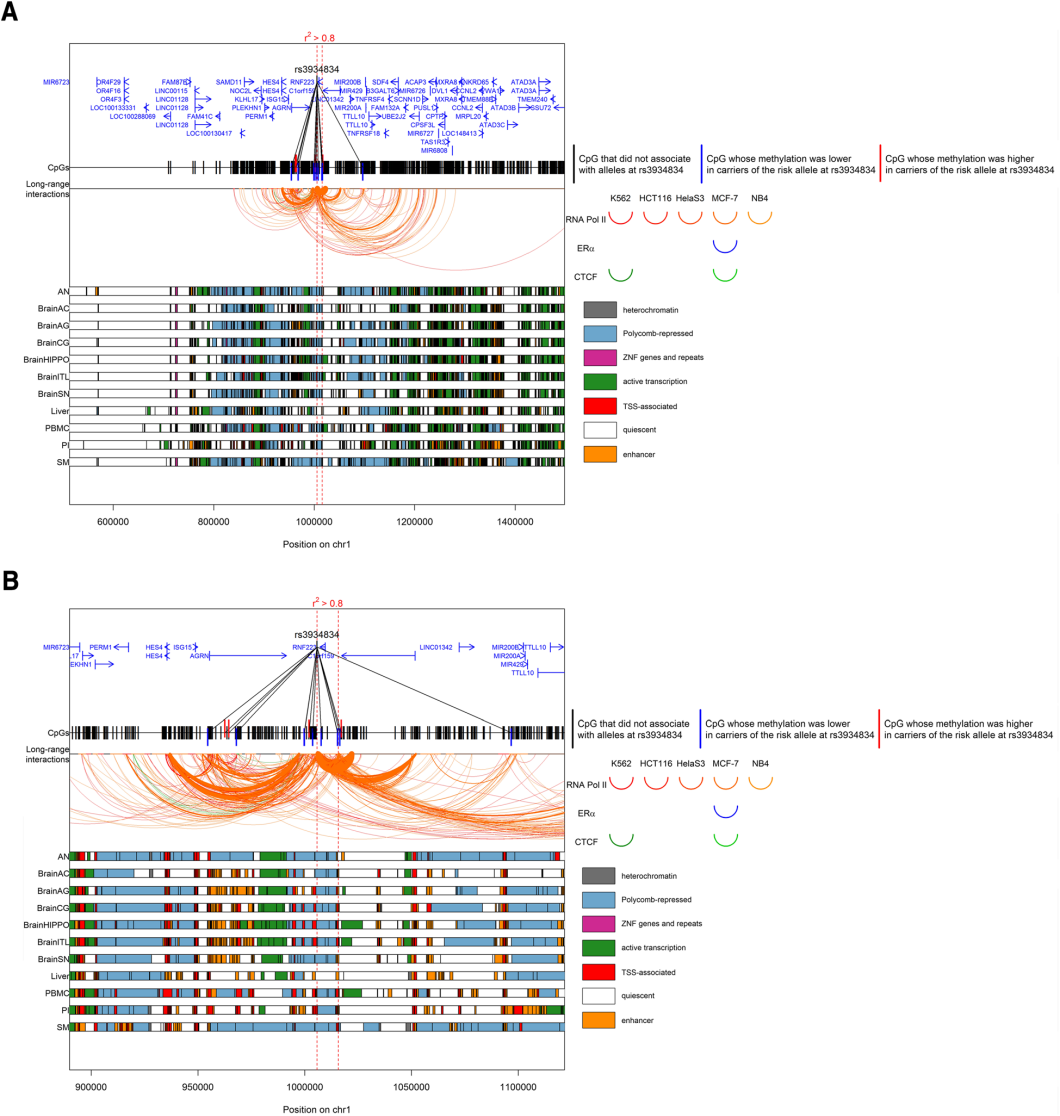
Genomic context of the CpGs associated with rs3934834. **a** The entire investigated region. **b** Zoom on the region surrounding rs3934834. Genomic positions of RefSeq genes and rs3934834 are displayed in the *top panel*. Within the two *vertical red dotted lines*, the LD r^2^ > 0.8. The positions of the tested CpGs are displayed. Long-range interactions as defined by ChIA-PET libraries from five cell lines using chromatin immunoprecipitation with antibodies targeting three transcription factors (Additional file 5) are displayed as arcs. For clarity of visualization, we chose to display only the long-range interactions of genomic regions containing associated CpGs. Two interacting genomic regions are represented by an arc that links them, and the thickness of the arc line is proportional to the strength of this interaction. The color of the arc corresponds to the target transcription factor and the shade of the color corresponds to the cell line: *red* for RNA polymerase II, *blue* for ERα, and *green* for CTCF. In the *bottom panel*, chromatin states in 11 tissues are displayed. Chromatin states were obtained using chromHMM prediction using data on seven histone marks (see “Methods”). The color of each band corresponds to a particular state. *AN* adipose nuclei, *BrainAC* brain anterior caudate, *BrainAG* brain angular gyrus, *BrainCG* brain cingulate gyrus, *BrainHIPPO* brain hippocampus, *BrainITL* brain inferior temporal lobe, *BrainSN* brain substantia nigra, *PBMC* peripheral blood mononuclear primary cells, *PI* pancreatic islets, *SM* skeletal muscle

**Fig. 7:**
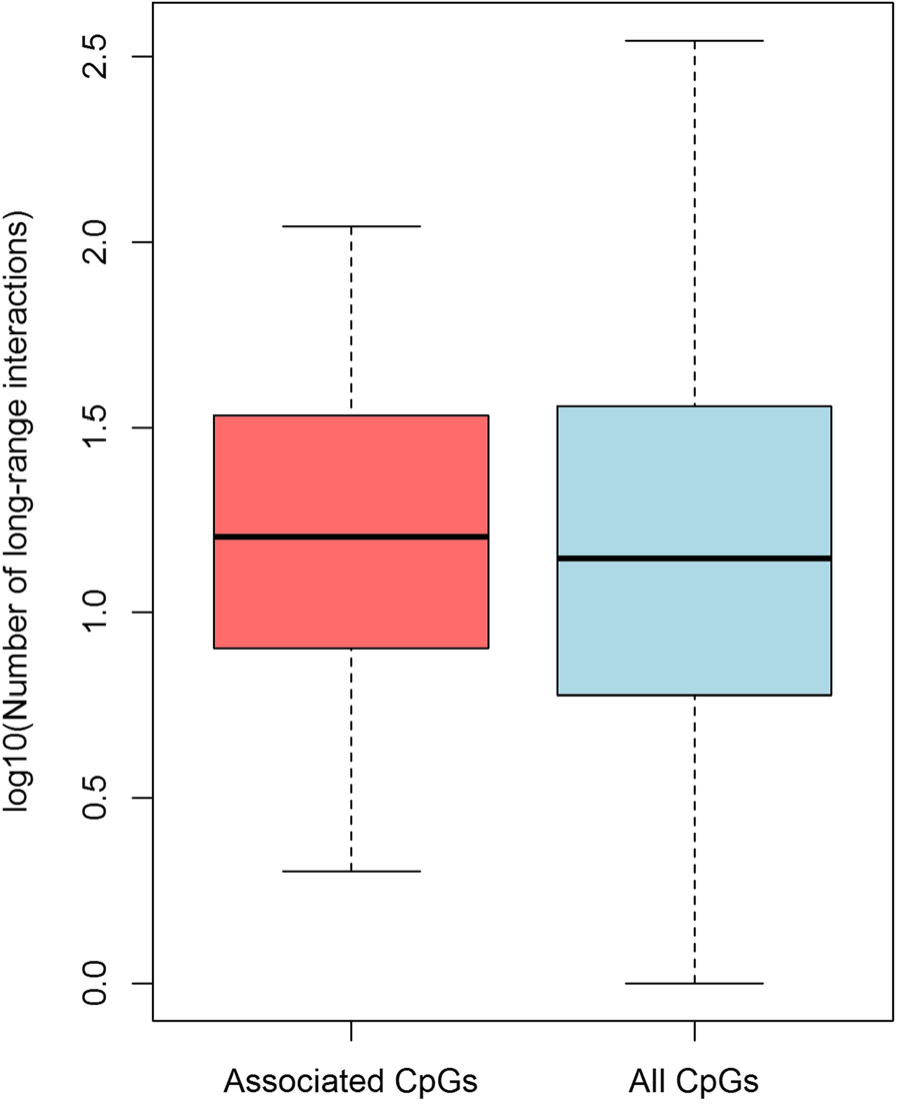
Distribution of the number of long-range interactions for associated versus all tested CpGs. For each associated and tested CpG, we counted the number of genomic regions containing the CpG that interacted with another genomic region. For clarity and because the number of interactions was skewed, we chose to display the log_10_(number of interactions)

Following the enrichment of associated CpGs at enhancers, we mapped all tested CpGs to long-range interactions as defined by ChIA-PET libraries from five cell lines and three transcription factors (Additional file 3) [63]. Of the 107 associated CpGs, 103 (96 %) were located in regions with at least one long-range interaction with another genomic region, and 73 of the 107 associated CpGs (68 %) were located in regions with at least one long-range interaction with a gene promoter (Additional file 4). For instance, five CpGs negatively associated with rs2444217 and located in enhancers in brain, PBMCs, liver, PIs, and SM showed long-range interactions with the same five gene promoters (Additional file 4; Fig. 5). Also, 6 of the 15 CpGs associated with alleles at rs3934834 were found to interact with no less than 11 promoters, and were in enhancers in PIs (Additional files 4 and 6; Fig. 6). The graphic results for all significant SNPs can be found in Additional file 7. Associated CpGs were not enriched in long-range interactions across all five cell lines and three target transcription factors (Mann–Whitney U-test *p* value > 0.05; Fig. 7).

### Associated CpGs are located in or show long-range interactions with the promoters of genes for which the corresponding SNPs are known eQTLs

We showed that some of the associated CpGs are located in gene promoters, and some are in regions showing putative long-range interactions with gene promoters. In order to make the link between SNP, methylation, and mRNA expression, we searched four eQTL databases (see “Methods”); we browsed all associated SNPs and SNPs in strong LD with them (with r^2^ > 0.8), and retrieved the genes for which they were eQTLs (Additional file 8). We found that associated CpGs are located in or show long-range interactions with the promoters of genes for which the corresponding SNPs are known eQTLs. For instance, rs10838738 is a known eQTL for several genes in blood, including *C1QTNF4*, *CELF1*, and *NUP160*. Interestingly, rs10838738 associated with three CpGs showing long-range interactions with *C1QTNF4* (Additional file 4), four CpGs showing long-range interactions with *CELF1* (Additional file 4), including three in enhancers in PBMCs (Additional file 6), and one CpG showing long-range interactions with *NUP160* (Additional file 4) that was in an enhancer in PBMCs (Additional file 4). Another example is rs713586, a known eQTL for *ADCY3* in blood and monocytes. rs713586 associated with a CpG located in the promoter of *ADCY3* (Additional file 4) that was also promoter-associated in PBMCs (Additional file 6).

### Genome-scale measurements are validated by bisulfite sequencing

We validated one of the tested CpGs (cg15576492) by bisulfite sequencing, using DNA from 17 individuals from the discovery study group who were homozygous for rs3934834 (six *A*/*A* and 11 *G*/*G*). Our criteria for choosing this site were the following: 1) significant association with risk alleles; 2) strongest association with risk alleles; 3) located in a gene promoter and/or having long-range interactions with a gene promoter. The correlation between methylation assessed by Illumina and bisulfite sequencing was good (Pearson’s correlation coefficient r = 0.68, *p* value = 0.0025; Additional file 9). Methylation in *A*/*A* was higher than methylation in *G*/*G*, but the methylation difference did not reach statistical significance (*p* value > 0.05), which could be explained by reduced statistical power (17 individuals).

### SNP–CpG associations might not be blood-specific

#### Four of the initial SNP–CpG associations in blood are replicated in skin fibroblasts

The open-access dataset of skin fibroblasts consists of DNA methylation data assessed with the Illumina HumanMethylation450 BeadChip and genotype data assessed with the Illumina Human1M-Duov3 DNA Analysis BeadChip. Thus, we had data to test 65 of the 107 significant SNP–CpG associations in skin fibroblasts (*n* = 62). Fourteen SNP–CpG associations had a raw *p* value < 0.05, and seven had a q value < 0.05, including four having a concordant effect sign with results obtained in blood (Additional file 10). Notably, genotypes at rs1011731 associated with methylation at cg13446689 (regression coefficient = 0.254, q value = 0.012).

#### The single SNP–methylation association tested in SAT and VAT was not significant

The SAT and VAT study group of 149 individuals (mostly overweight/obese) was used to test specifically the association between genotypes at rs1011731 and methylation at cg13446689, which was assessed by bisulfite sequencing. We chose to test this SNP–CpG pair because there was an association between cg13446689 and rs1011731 in both blood and fibroblasts, and because this study group had already been genotyped for rs1011731. There was no association between methylation at cg13446689 in VAT or SAT and genotypes at rs1011731 (*p* value > 0.05; Additional file 10).

#### The two SNP–methylation associations tested in cerebellum, frontal cortex, caudal pons, and temporal cortex were not significant

The open-access dataset of four brain regions consists of DNA methylation assayed using the Illumina HumanMethylation27 BeadChip, and genotype data assessed with the Illumina Human1M-Duov3 DNA Analysis BeadChip. Thus, we had data to test two of the 107 associated CpGs (cg05585544 and cg11385473). There was no association between genotypes at rs10838738 and methylation at cg05585544 in any of the four brain regions; there was no association between genotypes at rs652722 and methylation at cg11385473 in any of the four brain regions (*p* values > 0.05; Additional file 10).

## Discussion

Our findings suggest that carriers of obesity-associated risk alleles display complex alterations of the gene regulatory landscape. We find that obesity-associated SNPs can be linked to DNA methylation levels in several proximal locations, which implies that they may affect multiple genes. These SNPs associated with proximal DNA methylation levels in whole blood of healthy individuals, but these associations might not be blood-specific. Interestingly, several obesity-associated SNPs associated with CpGs that were in the promoters of genes known to participate in the pathogenesis of obesity, or were located in regions that interact with such genes. In addition, associated CpGs were enriched in enhancers in blood, which highlights their potential in gene regulation.

It is well established that DNA methylation levels correlate with the presence of specific alleles at nearby SNPs [25–37], such as Grundberg et al. [29], who found that 28 % of CpGs were associated with SNPs within 100 kb in adipose tissue. If we restrict our analysis to 100 kb, we find 103 SNP–CpG associations at a q value < 0.05, corresponding to 27 unique SNPs (52 % of tested SNPs). It would be difficult to assess whether this percentage is particularly high, but the present study shows that obesity-associated SNPs discovered in GWASs may mediate their effect through alterations of the regulation of transcription. Indeed, the global results for each significant SNP display fascinating patterns. Several obesity-associated SNPs may affect “transcription factories”, clusters of gene promoters and their enhancers that interact in three-dimensional space and are brought together by DNA-binding proteins such as CTCF [70]. The most striking example is rs7498665 since the 12 CpGs associated with this SNP are located in ten distinct gene promoters. rs3888190, one of the top loci of the most recent BMI GWAS [11], is in perfect LD with rs7498665 (r^2^ = 1) and is known to be an eQTL for five of these ten gene promoters (*APOBR* [71], *SH2B1* [71], *SULT1A2* [72], *ATXN2L* [11], and *TUFM* [11]). Another interesting example is rs10838738, which associated with three CpGs showing long-range interactions with *C1QTNF4*, four CpGs showing long-range interactions with *CELF1*, and one CpG showing long-range interactions with *NUP160*. rs10838738 is a known eQTL for these three genes in blood [64, 66]. Thus, our results suggest that the effect of obesity-associated SNPs may be mediated by multiple and quite distant genes, as illustrated by three of our investigated SNPs (rs3934834, rs2287019, and rs7498665) that associated with CpGs interacting with no less than 15 promoters (Additional files 4 and 7). This underlines the importance for a rational and inclusive selection process for candidate genes for GWAS hits rather than the common practice of only focusing on the closest gene.

At a more detailed level, patterns of DNA methylation at specific CpGs between carriers and non-carriers of risk alleles were consistent with previous studies. Alleles at rs713586 explained 54 % of the variance in methylation at cg01884057, with an increase of almost 10 % methylation for each risk allele. The very same pattern was also found in adipose tissue in another study [29]. More interestingly, some patterns of DNA methylation between carriers and non-carriers of risk alleles was consistent with what is known about these genes and obesity. For instance, *MIR148A* is upregulated during normal adipogenesis but downregulated in obese adipocytes [73], and its expression is regulated by DNA methylation at its CpG island [74]. Consistently, carriers of the risk allele at rs1055144 had higher methylation levels in the promoter of *MIR148A*. Also, carriers of the risk allele at rs10838738 had lower methylation in the promoter of *PTPMT1*, a gene that codes for a mitochondrial phosphatase whose inhibition lowers glucose concentration [75] and a suggested drug target for treatment of type II diabetes [76]. Last but not least, three of the associated CpGs were located within two of the numerous promoters of *BDNF*, which encodes a neurotrophin that plays several roles in regulating energy homeostasis [77]. It is suggested that *BDNF* is finely regulated by DNA methylation and histone modifications [78, 79], and differential *BDNF* transcripts are expressed at different time points and in different cellular compartments [79]. Carriers of the risk allele at rs10767664 had higher methylation in the pII promoter of *BDNF*, and lower methylation in the pVI promoter of *BDNF*. However, the roles of specific *BDNF* promoters in obesity remain unexplored. Also, the SNPs may affect other genes linked to obesity: NR1H3, a member of the liver X receptors that regulate cholesterol catabolism [80] and expressed during adipose tissue remodeling [81]; PACSIN3, a kinase that induces glucose uptake by adipocytes [82]; LGR4, a G protein-coupled receptor whose ablation potentiates the white-to-brown fat transition [83]; POMC, a peptide that decreases food intake and increases energy expenditure [84]; CLN3 and ITH4, two proteins positively associated with obesity [85, 86]; and the developmental genes *HOTAIR* and *HOXC11*, responsible for differential fat accumulation between upper and lower body, and under epigenetic control [85, 87].

Our study aimed at unraveling the molecular effects of body mass-associated genetic variants on chromatin structure, with a special focus on DNA methylation. We benefited from a large sample size for the discovery analysis (*n* = 355) and from the use of a large battery of open access datasets to map associated CpGs to meaningful genomic annotations, such as promoters, predicted and in vivo transcribed enhancers, and long-range interactions. In addition, we tested the tissue-specificity of 65 of the initial associations in skin fibroblasts, two of the initial associations in four brain regions, and one in SAT and VAT. It is possible that some of the associations discovered in blood are limited to this tissue, or that unmeasured environmental factors such as smoking, diet, physical activity, and tissue-specific molecular factors impacted DNA methylation at the measured CpG sites and confounded our results. It should be noted, however, that we could not test all of the 107 initial associations in the replication samples, and the sample sizes of the replication samples were smaller than the discovery study group. Analysis of statistical power (probability of detecting a “true” effect when it exists) suggests that we have a high probability of replicating our results in the VAT and SAT replication samples, where power was 95 %. In contrast, power was only 23–25 % for cg11385473 and 42–47 % for cg05585544 for the brain replication samples and 39 % on average for the skin fibroblast replication samples, which implies that we are likely unable to replicate our results due to too small sample groups for these conditions. Besides, pan-tissue SNP–CpG associations are consistent with a genome-wide study where genotype-dependent methylation differences between blood and brain were associated, making genetic influence on DNA methylation in blood relevant for other tissues [26]. Finally, it should be kept in mind that the probes of the methylation array used in this study (Illumina 450 k) are enriched in CpG islands, gene promoters, and gene regions; it is thus possible that we missed important CpGs linked to obesity-associated SNPs.

In the paradigm of genetics–epigenetics–environment relationships, it is still unknown whether obesity-associated SNPs directly cause differential DNA methylation at genes and enhancers that contribute to the pathogenesis of obesity, or if the observed differential methylation levels are merely a consequence of a modified gene regulation caused by the presence of risk alleles at obesity-associated SNPs. In a recent review on the function and information content of DNA methylation, DNA methylation is thought to have both an active and passive role in gene regulation, and it seems to be highly contextual [88]. In particular, it has been proposed that mutations within regulatory regions affect binding of transcription factors, which in turn influence DNA methylation [88]. If DNA methylation does not necessarily actively impact on gene regulation, it is at least an informative marker of the underlying regulatory activity. Therefore, the differential methylation observed in carriers of risk alleles at obesity-associated SNPs in our study likely reflects allele-specific effects on gene regulatory mechanisms.

## Conclusions

In this study we report strong associations between obesity-associated SNPs discovered in GWASs and methylation levels at proximal CpG sites. The methylation sites associated with alleles at obesity-associated SNPs were enriched in enhancers in PBMCs, and some of these sites were located in the promoters of genes, or were located in regions showing long-range interactions with established roles in appetite regulation as well as regulation of body mass. We also found indications that some of these genotype–methylation associations exist in different tissues. This study has implications for understanding how obesity-associated variants mediate their effects. Further studies are needed to unravel the mechanisms that govern the interplay between genetic variants and the activity of functional DNA elements.

## Additional files

The following additional data are available with the online version of this paper. Additional file 1 is a is a table describing the 52 investigated SNPs. Additional file 2 is a heatmap showing the top CpG sites associated with blood cell type surrogates (principal components), evaluated in purified human leukocyte subtype methylation data sets. Additional file 3 is a table listing the cell lines and target transcription factors of the ChIA-PET libraries. Additional file 4 is a table describing the 107 associated CpGs found in blood and their annotation. Additional file 5 is a PCA showing the two discovery study samples on the first three principal components, using only the most variable autosomal CpG sites. Additional file 6 is a table showing the chromatin state at the genomic position of the 107 associated CpG, in 11 tissues. Additional file 7 is a figure showing the genomic context of the CpGs associated with the 28 significant SNPs. Additional file 8 is a table showing the eQTLs found in four eQTL databases for each of the 28 significant SNPs. Additional file 9 is a figure showing the correlations between Illumina 450 K and pyrosequence analysis of cg15576492. Additional file 10 is a table summarizing the replication of the 107 SNP-CpG associations found in blood. Additional file 1:
**Description of the 52 investigated SNPs.** SNPs in bold are SNPs for which significant associations with DNA methylation were found. *Number in parenthesis = number of individuals with missing genotypes. (DOCX 108 kb) Additional file 2:
**Top CpG sites associated with blood cell type surrogates (principal components), evaluated in purified human leukocyte subtype methylation data sets.** (TIFF 26367 kb) Additional file 3:
**Cell lines and target transcription factors of the ChIA-PET libraries.** ^#^Cell types are described in ENCODE project [89]. *Target transcription factors and their corresponding antibodies are described in the ENCODE project [90]. (DOCX 11 kb) Additional file 4:
**Description of the 107 associated CpGs found in blood and their annotation.** *Coefficient of the linear model associated with the obesity-associated SNP: positive for increased methylation with presence of risk allele; coefficients are calculated using M values. (DOCX 38 kb) Additional file 5:
**Principal component analysis of the two study groups (** ***n*** **= 355) on the first three principal components, using only the most variable autosomal CpG sites.** *Red dots* are individuals from study sub-group 1 (*n* = 130), *black dots* are individuals from study sub-group 2 (*n* = 225). (TIFF 26367 kb) Additional file 6:
**Chromatin states at the genomic position of the 107 CpGs, in the 11 investigated tissues.** *AN* adipose nuclei, *BrainAC* brain anterior caudate, *BrainAG* brain angular gyrus, *BrainCG* brain cingulate gyrus, *BrainHIPPO* brain hippocampus, *BrainITL* brain inferior temporal lobe, *BrainSN* brain substantia nigra, *PBMC* peripheral blood mononuclear primary cells, *PI* pancreatic islets, *SM* skeletal muscle. (DOCX 36 kb) Additional file 7:
**Genomic context of the CpGs associated with the significant SNPs.** Each plot corresponds to a SNP for which associations with DNA methylation were found (28 plots in total). Genomic positions of RefSeq genes and the obesity-related SNP are displayed in the *top panel*. Within the two *vertical red dotted lines*, the linkage disequilibrium r^2^ > 0.8. The positions of the tested CpGs are displayed. Long-range interactions as defined by ChIA-PET libraries from five cell lines using chromatin immunoprecipitation with antibodies targeting three transcription factors (Additional file 4) are displayed as arcs. For clarity of visualization, we chose to display only the long-range interactions of genomic regions containing associated CpGs. Two interacting genomic regions are represented by an arc that links them, and the thickness of the arc line is proportional to the strength of this interaction. The color of the arc corresponds to the target transcription factor and the shade of the color corresponds to the cell line: *red* for RNA polymerase II, *blue* for ERα, and *green* for CTCF. In the *bottom panel*, chromatin states in 11 tissues are displayed. Chromatin states were obtained using chromHMM prediction using data on seven histone marks (see “Methods”). The color of each band corresponds to a particular state. *AN* adipose nuclei, *BrainAC* brain anterior caudate, *BrainAG* brain angular gyrus, *BrainCG* brain cingulate gyrus, *BrainHIPPO* brain hippocampus, *BrainITL* brain inferior temporal lobe, *BrainSN* brain substantia nigra, *PBMC* peripheral blood mononuclear primary cells, *PI* pancreatic islets, *SM* skeletal muscle. (TIFF 9613 kb) Additional file 8:
**eQTLs found in four eQTL databases for each of the significant 28 SNPs.** ^1^Investigated tissues: subcutaneous adipose tissue, aorta artery, tibial artery, esophagus mucosa, esophagus muscularis, heart left ventricle, lung, skeletal muscle, tibial nerve, sun-exposed skin, lower leg, stomach, thyroid, whole blood. ^2^Investigated tissues/cell lines: lymphoblastoid cell line (LCL), liver, monocytes, fibroblasts, T cells, brain cortex. ^3^Investigated tissues/cell lines: lymphoblastoid cell line (LCL), liver, brain cerebellum, brain frontal cortex, brain temporal cortex, brain pons. (DOCX 25 kb) Additional file 9:
**Correlations between Illumina 450 K and pyrosequence analysis of cg15576492.** Methylation at cg15576492, as determined by the Illumina 450 k Chip and expressed as β value, is plotted against methylation at cg15576492, as determined by pyrosequencing and expressed as β value (*n* = 17). (TIFF 12920 kb) Additional file 10:
**Replication of the 107 SNP-CpG associations found in blood.** *Coefficient of the linear model associated with the obesity-associated SNP: positive for increased methylation with presence of risk allele; coefficients are calculated using M values. Coefficients with a hash symbol correspond to associations with raw *p* value < 0.05 and coefficients in bold correspond to associations with q value < 0.05. (DOCX 31 kb)

## Abbreviations

AN: adipose nuclei
BMI: body mass index
BrainAC: brain anterior caudate
BrainAG: brain angular gyrus
BrainCG: brain cingulate gyrus
BrainHIPPO: brain hippocampus
BrainITL: brain inferior temporal lobe
BrainSN: brain substantia nigra
CAGE: cap-analysis of gene expression
ChIA-PET: chromatin interaction analysis by paired-end tag sequencing
eQTL: expression quantitative trait locus
GEO: Gene Expression Omnibus
GWAS: genome-wide association study
LD: linkage disequilibrium
meQTL: methylation quantitative trait locus
PBMC: peripheral blood mononuclear primary cell
PCA: principal component analysis
PCR: polymerase chain reaction
PI: pancreatic islet
QTL: quantitative trait locus
SAT: subcutaneous adipose tissue
SM: skeletal muscle
SNP: single nucleotide polymorphism
TSS: transcription start site
UCSC: University of California, Santa Cruz
VAT: visceral adipose tissue
