# Supplementary material for: Many obesity-associated SNPs strongly associate with DNA methylation changes at proximal promoters and enhancers
Source: Genome Med. 2015 Oct 8;7:103. doi: 10.1186/s13073-015-0225-4 (PMC4599317; doi:10.1186/s13073-015-0225-4)
Supplement: Additional file 1: — Description of the 52 investigated SNPs. SNPs in bold are SNPs for which significant associations with DNA methylation were found. *Number in parenthesis = number of individuals with missing genotypes. (DOCX 108 kb) [file 13073_2015_225_MOESM1_ESM.docx]

| SNP | Genomic location (hg19) | Closest gene | Risk allele | Obesity-related trait | Original study | Number of individuals of the discovery cohorts with genotypes* | Genotype frequencies in the discovery cohorts | Hardy-Weinberg p-value | Number of tested probes | Number of significant probes |
| --- | --- | --- | --- | --- | --- | --- | --- | --- | --- | --- |
| **rs1011731** | chr1:172346548 | *DNM3* | G | BMI-adjusted WHR | Heid et al., 2010 [18] | 355 | A/A: 94(27%); A/G: 175(49%); G/G: 86(24%) | 0.83 | 63 | 1 |
| **rs10150332** | chr14:79936964 | *NRXN3* | C | BMI | Speliotes et al., 2011 [2] | 355 | T/T: 224(63%); T/C: 113(32%); C/C: 18(5.1%) | 0.43 | 37 | 1 |
| rs10195252 | chr2:165513091 | *COBLL1* | T | Circulating TG | Teslovich et al., 2010 [15] | 355 | C/C: 61(17%); C/T: 171(48%); T/T: 123(35%) | 0.91 | 30 | 0 |
| rs10458787 | chr10:4655565 | *LINC00704* | G | BMI | Liu et al., 2010 [5] | 354 (1) | A/A: 23(6.5%); A/G: 111(31%); G/G: 220(62%) | 0.09 | 60 | 0 |
| **rs1055144** | chr7:25871109 | *RNU6-16P* | T | BMI-adjusted WHR | Heid et al., 2010 [18] | 355 | C/C: 254(72%); C/T: 87(25%); T/T: 14(3.9%) | 0.077 | 129 | 6 |
| **rs10767664** | chr11:27725986 | *BDNF* | A | BMI | Speliotes et al., 2011 [2] | 355 | T/T: 12(3.4%); T/A: 115(32%); A/A: 228(64%) | 0.59 | 145 | 4 |
| **rs10769908** | chr11:8484089 | *STK33* | C | BMI | Willer et al., 2009 [1] | 355 | T/T: 95(27%); T/C: 176(50%); C/C: 84(24%) | 0.92 | 207 | 4 |
| rs10783050 | chr1:97037083 | *PTBP2* | C | BMI | Thorleifsson et al., 2009 [4] | 355 | T/T: 158(45%); T/C: 168(47%); C/C: 29(8.2%) | 0.11 | 25 | 0 |
| **rs10838738** | chr11:47663049 | *MTCH2* | G | BMI | Willer et al., 2009 [1] | 355 | A/A: 161(45%); A/G: 148(42%); G/G: 46(13%) | 0.19 | 312 | 8 |
| rs10993160 | chr9:97068926 | *ZNF169* | A | BMI | Okada et al., 2012 [3] | 355 | G/G: 0(0%); G/A: 20(5.6%); A/A: 335(94%) | 1 | 88 | 0 |
| rs11142387 | chr9:72998332 | *KLF9* | C | BMI | Okada et al., 2012 [3] | 352 (3) | A/A: 85(24%); A/C: 172(48%); C/C: 95(27%) | 0.67 | 34 | 0 |
| **rs1152846** | chr3:188420897 | *LPP* | G | BMI, weight | Johansson et al., 2010 [6] | 355 | A/A: 19(5.4%); A/G: 146(41%); G/G: 190(54%) | 0.21 | 72 | 1 |
| **rs12517906** | chr5:180170819 | *OR2Y1* | T | Weight and BMI in females | Johansson et al., 2010 [6] | 355 | C/C: 262(74%); C/T: 86(24%); T/T: 7(2%) | 1 | 333 | 2 |
| rs12597579 | chr16:20257867 | *GP2* | C | BMI | Wen et al., 2012 [8] | 354 (1) | T/T: 2(0.56%); T/C: 41(12%); C/C: 311(88%) | 0.64 | 93 | 0 |
| rs1294421 | chr6:6743149 | *LY86* | G | BMI-adjusted WHR | Heid et al., 2010 [18] | 355 | T/T: 53(15%); T/G: 165(46%); G/G: 137(39%) | 0.82 | 118 | 0 |
| rs13107325 | chr4:103188709 | *SLC39A8* | T | Circulating HDL | Teslovich et al., 2010 [15] | 355 | C/C: 329(93%); C/T: 26(7.3%); T/T: 0(0%) | 1 | 69 | 0 |
| **rs1443512** | chr12:54342684 | *HOCX13* | A | BMI-adjusted WHR | Heid et al., 2010 [18] | 298 (57) | C/C: 151(43%); C/A: 126(35%); A/A: 21(5.9%) | 0.57 | 581 | 4 |
| rs1514175 | chr1:74991644 | *TNNI3K, FPGT-TNNI3K* | A | BMI | Speliotes et al., 2011 [2] | 355 | G/G: 96(27%); G/A: 193(54%); A/A: 66(19%) | 0.087 | 42 | 0 |
| **rs17782313** | chr18:57851097 | *MC4R* | C | Fat mass, weight and risk of obesity | Loos et al., 2008 [12] | 355 | T/T: 194(55%); T/C: 140(39%); C/C: 21(5.9%) | 0.58 | 24 | 1 |
| **rs1878047** | chr19:51773802 | *SIGLECL1* | G | BMI | Johansson et al., 2010 [6] | 355 | A/A: 136(38%); A/G: 174(49%); G/G: 45(13%) | 0.43 | 348 | 3 |
| **rs1927702** | chr9:15986716 | *CCDC171* | G | BMI in females | Johansson et al., 2010 [6] | 355 | A/A: 118(33%); A/G: 164(46%); G/G: 73(21%) | 0.28 | 25 | 1 |
| **rs206936** | chr6:34302869 | *NUDT3, RPS10-NUDT3* | G | BMI | Speliotes et al., 2011 [2] | 355 | A/A: 218(61%); A/G: 114(32%); G/G: 23(6.5%) | 0.13 | 282 | 1 |
| **rs2112347** | chr5:75015242 | *POC5* | T | BMI | Speliotes et al., 2011 [2] | 352 (3) | G/G: 42(12%); G/T: 180(51%); T/T: 130(37%) | 0.11 | 84 | 1 |
| rs2145270 | chr20:6621685 | *CASC20* | T | BMI | Willer et al., 2009 [1] | 355 | C/C: 43(12%); C/T: 168(47%); T/T: 144(41%) | 0.64 | 16 | 0 |
| rs2206734 | chr6:20694884 | *CDKAL1* | C | BMI | Okada et al., 2012 [3] | 353 (2) | T/T: 10(2.8%); T/C: 100(28%); C/C: 243(68%) | 1 | 72 | 0 |
| **rs2241423** | chr15:68086838 | *MAP2K5* | G | BMI | Speliotes et al., 2011 [2] | 355 | A/A: 17(4.8%); A/G: 106(30%); G/G: 232(65%) | 0.31 | 155 | 6 |
| **rs2287019** | chr19:46202172 | *QPCTL* | C | BMI | Speliotes et al., 2011 [2] | 355 | T/T: 12(3.4%); T/C: 124(35%); C/C: 219(62%) | 0.33 | 483 | 6 |
| rs2383393 | chr4:180664653 | *LINC00290* | G | BMI in males | Johansson et al., 2010 [6] | 355 | A/A: 52(15%); A/G: 173(49%); G/G: 130(37%) | 0.74 | 10 | 0 |
| **rs2444217** | chr16:4038387 | *ADCY9* | A | BMI | Speliotes et al., 2011 [2] | 355 | G/G: 105(30%); G/A: 170(48%); A/A: 80(23%) | 0.52 | 368 | 4 |
| rs261967 | chr5:95850250 | *LOC101929710* | C | BMI | Wen et al., 2012 [8] | 355 | A/A: 102(29%); A/C: 169(48%); C/C: 84(24%) | 0.4 | 113 | 0 |
| **rs2815752** | chr1:72812440 | *NEGR1* | A | BMI | Cotsapas et al., 2009 [7] | 355 | G/G: 48(14%); G/A: 174(49%); A/A: 133(37%) | 0.5 | 19 | 1 |
| **rs3934834** | chr1:1005806 | *RNF223* | G | BMI | Johansson et al., 2010 [6] | 355 | A/A: 14(3.9%); A/G: 116(33%); G/G: 225(63%) | 1 | 889 | 15 |
| rs4377469 | chr3:42303074 | *CCK* | T | BMI | Okada et al., 2012 [3] | 355 | G/G: 5(1.4%); G/T: 87(25%); T/T: 263(74%) | 0.65 | 180 | 0 |
| rs4823006 | chr22:29451671 | *ZNRF3* | A | BMI-adjusted WHR | Heid et al., 2010 [18] | 355 | G/G: 66(19%); G/A: 181(51%); A/A: 108(30%) | 0.59 | 184 | 0 |
| rs516636 | chr1:177855517 | *SEC16B* | A | BMI | Okada et al., 2012 [3] | 355 | C/C: 229(65%); C/A: 116(33%); A/A: 10(2.8%) | 0.39 | 48 | 0 |
| **rs652722** | chr11:31905534 | *DKFZp686K1684* | C | BMI | Wen et al., 2012 [8] | 355 | T/T: 27(7.6%); T/C: 129(36%); C/C: 199(56%) | 0.33 | 169 | 1 |
| **rs6784615** | chr3:52506426 | *NISCH* | T | BMI-adjusted WHR | Heid et al., 2010 [18] | 355 | C/C: 0(0%); C/T: 30(8.5%); T/T: 325(92%) | 1 | 369 | 5 |
| rs6794092 | chr3:171558344 | *TMEM212* | G | BMI | Ng et al., 2012 [9] | 355 | A/A: 1(0.28%); A/G: 8(2.3%); G/G: 346(97%) | 0.062 | 107 | 0 |
| **rs6795735** | chr3:64705365 | *ADAMTS9-AS2* | C | BMI-adjusted WHR | Heid et al., 2010 [18] | 355 | T/T: 70(20%); T/C: 167(47%); C/C: 118(33%) | 0.45 | 82 | 1 |
| **rs6861681** | chr5:173362458 | *CPEB4* | A | BMI-adjusted WHR | Heid et al., 2010 [18] | 333 (22) | G/G: 155(44%); G/A: 138(39%); A/A: 40(11%) | 0.32 | 126 | 4 |
| rs6905288 | chr6:43758873 | *VEGFA* | A | BMI-adjusted WHR | Heid et al., 2010 [18] | 355 | G/G: 52(15%); G/A: 186(52%); A/A: 117(33%) | 0.13 | 311 | 0 |
| **rs713586** | chr2:25158008 | *DNAJC27* | C | BMI | Speliotes et al., 2011 [2] | 353 (2) | T/T: 101(28%); T/C: 160(45%); C/C: 92(26%) | 0.088 | 225 | 8 |
| rs7138803 | chr12:50247468 | *BCDIN3D* | A | WC | Heard-Costa et al., 2009 [16] | 355 | G/G: 125(35%); G/A: 172(48%); A/A: 58(16%) | 1 | 307 | 0 |
| **rs718314** | chr12:26453283 | *ITPR2* | G | BMI-adjusted WHR | Heid et al., 2010 [18] | 355 | A/A: 184(52%); A/G: 145(41%); G/G: 26(7.3%) | 0.79 | 83 | 1 |
| **rs7481311** | chr11:27583129 | *BDNF-AS* | T | BMI | Thorleifsson et al., 2009 [4] | 355 | C/C: 202(57%); C/T: 129(36%); T/T: 24(6.8%) | 0.57 | 134 | 3 |
| **rs7498665** | chr16:28883241 | *SH2B1* | G | Serum leptin, total fat, WC | Jamshidi et al., 2007 [13] | 355 | A/A: 123(35%); A/G: 164(46%); G/G: 68(19%) | 0.33 | 229 | 12 |
| rs7647305 | chr3:185834290 | *ETV5* | C | BMI | Cotsapas et al., 2009 [14] | 355 | T/T: 8(2.3%); T/C: 121(34%); C/C: 226(64%) | 0.089 | 148 | 0 |
| rs7832552 | chr8:110115676 | *TRHR* | C | Lean body mass | Liu et al., 2009 [15] | 355 | T/T: 47(13%); T/C: 159(45%); C/C: 149(42%) | 0.64 | 63 | 0 |
| rs824931 | chr2:222801699 | *PAX3* | G | BMI | Johansson et al., 2010 [6] | 355 | A/A: 144(41%); A/G: 165(46%); G/G: 46(13%) | 1 | 146 | 0 |
| rs887912 | chr2:59302877 | *LINC01122* | T | BMI | Speliotes et al., 2011 [2] | 355 | C/C: 198(56%); C/T: 133(37%); T/T: 24(6.8%) | 0.78 | 24 | 0 |
| **rs984222** | chr1:119503843 | *TBX15* | G | BMI-adjusted WHR | Heid et al., 2010 [18] | 355 | C/C: 37(10%); C/G: 169(48%); G/G: 149(42%) | 0.35 | 118 | 2 |
| rs9939609 | chr16:53820527 | *FTO* | A | BMI | Field et al., 2007 [10] | 355 | T/T: 136(38%); T/A: 164(46%); A/A: 55(15%) | 0.65 | 106 | 0 |
