# Supplementary figures and images for: Many obesity-associated SNPs strongly associate with DNA methylation changes at proximal promoters and enhancers
Source: Genome Med. 2015 Oct 8;7:103. doi: 10.1186/s13073-015-0225-4 (PMC4599317; doi:10.1186/s13073-015-0225-4)

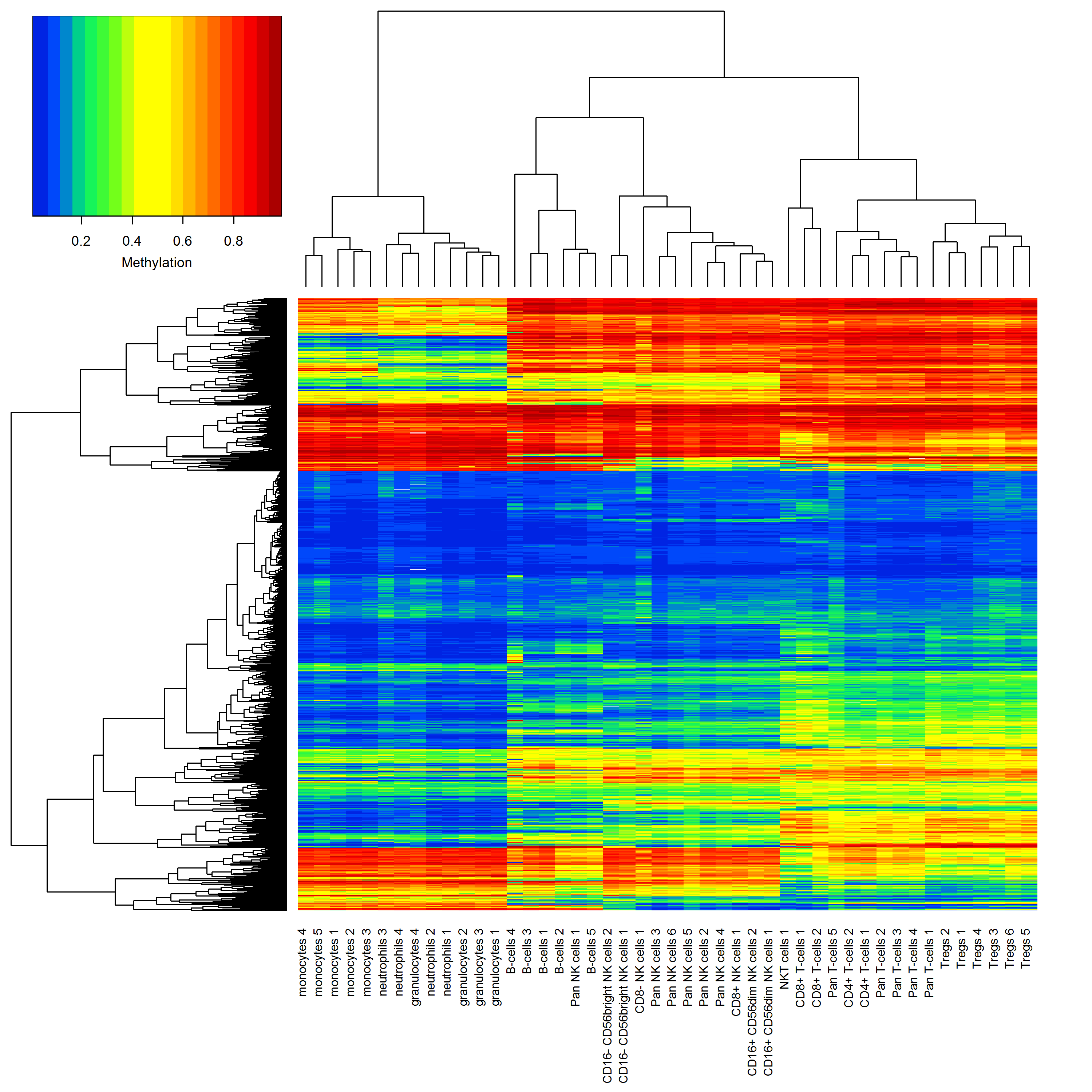

Supplement: Additional file 2: — Top CpG sites associated with blood cell type surrogates (principal components), evaluated in purified human leukocyte subtype methylation data sets. (TIFF 26367 kb) [file 13073_2015_225_MOESM2_ESM.tif]

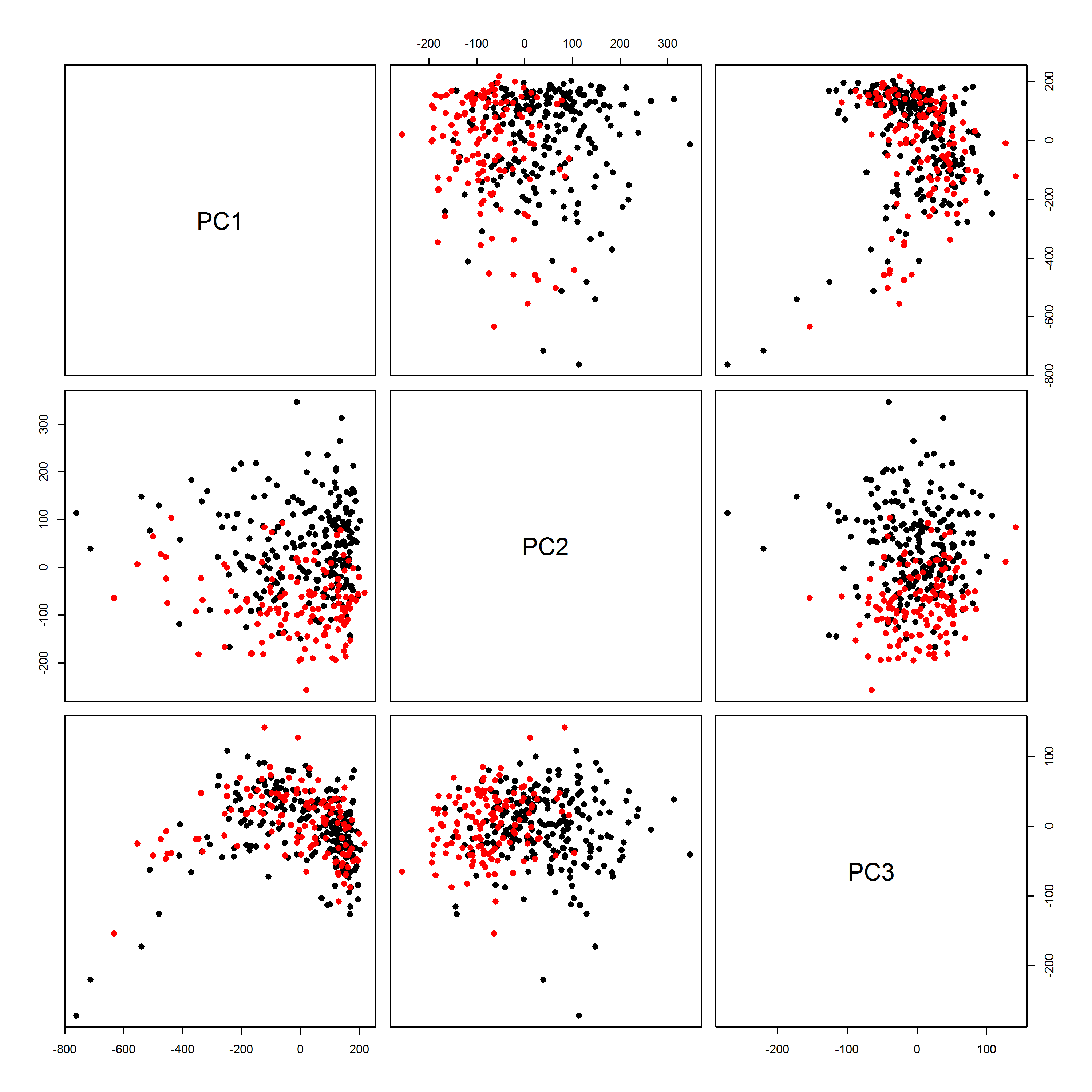

Supplement: Additional file 5: — Principal component analysis of the two study groups ( n = 355) on the first three principal components, using only the most variable autosomal CpG sites. Red dots are individuals from study sub-group 1 (n = 130), black dots are individuals from study sub-group 2 (n = 225). (TIFF 26367 kb) [file 13073_2015_225_MOESM5_ESM.tif]

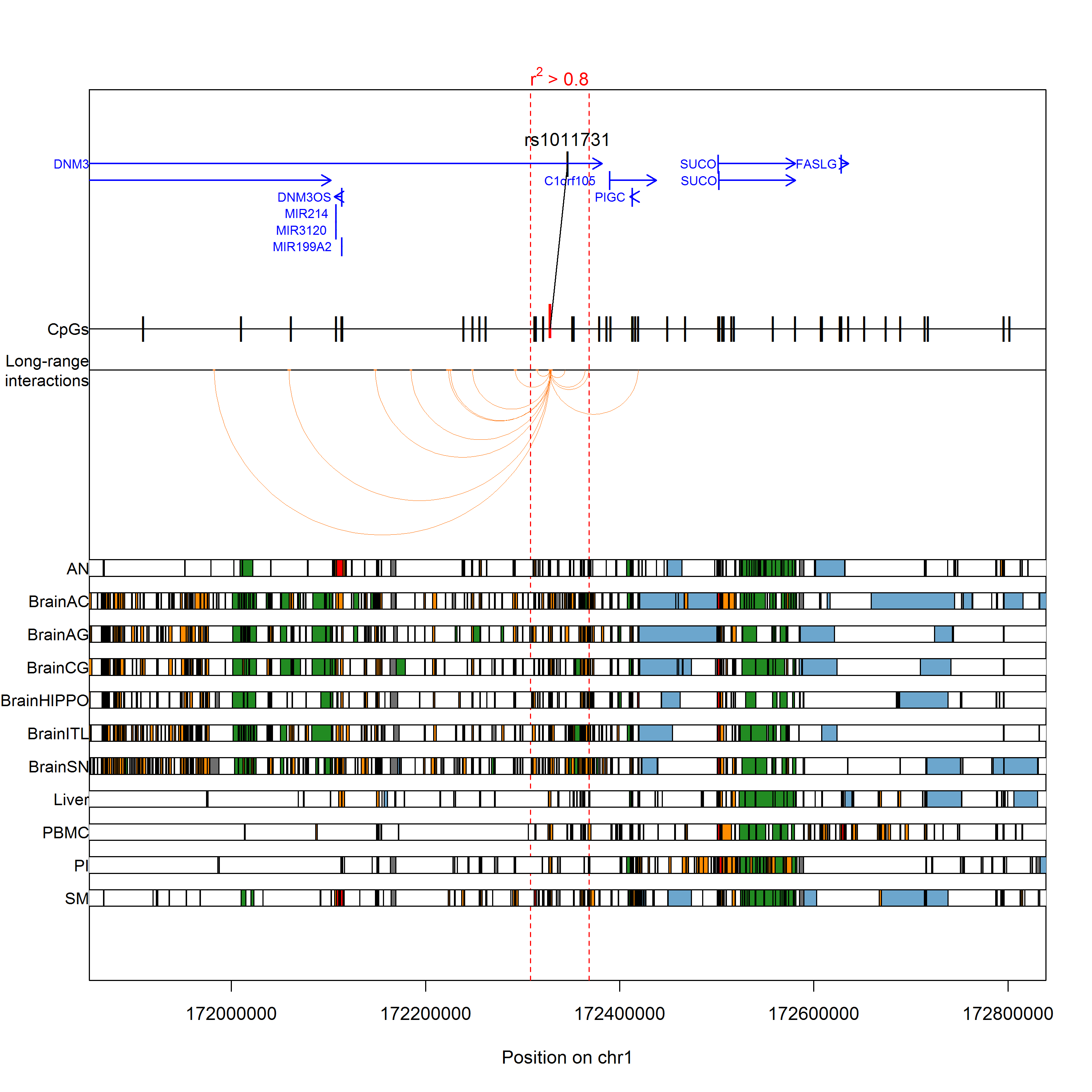

Supplement: Additional file 7: — Genomic context of the CpGs associated with the significant SNPs. Each plot corresponds to a SNP for which associations with DNA methylation were found (28 plots in total). Genomic positions of RefSeq genes and the obesity-related SNP are displayed in the top panel. Within the two vertical red dotted lines, the linkage disequilibrium r2 > 0.8. The positions of the tested CpGs are displayed. Long-range interactions as defined by ChIA-PET libraries from five cell lines using chromatin immunoprecipitation with antibodies targeting three transcription factors (Additional file 4) are displayed as arcs. For clarity of visualization, we chose to display only the long-range interactions of genomic regions containing associated CpGs. Two interacting genomic regions are represented by an arc that links them, and the thickness of the arc line is proportional to the strength of this interaction. The color of the arc corresponds to the target transcription factor and the shade of the color corresponds to the cell line: red for RNA polymerase II, blue for ERα, and green for CTCF. In the bottom panel, chromatin states in 11 tissues are displayed. Chromatin states were obtained using chromHMM prediction using data on seven histone marks (see “Methods”). The color of each band corresponds to a particular state. AN adipose nuclei, BrainAC brain anterior caudate, BrainAG brain angular gyrus, BrainCG brain cingulate gyrus, BrainHIPPO brain hippocampus, BrainITL brain inferior temporal lobe, BrainSN brain substantia nigra, PBMC peripheral blood mononuclear primary cells, PI pancreatic islets, SM skeletal muscle. (TIFF 9613 kb) [file 13073_2015_225_MOESM7_ESM.tif]

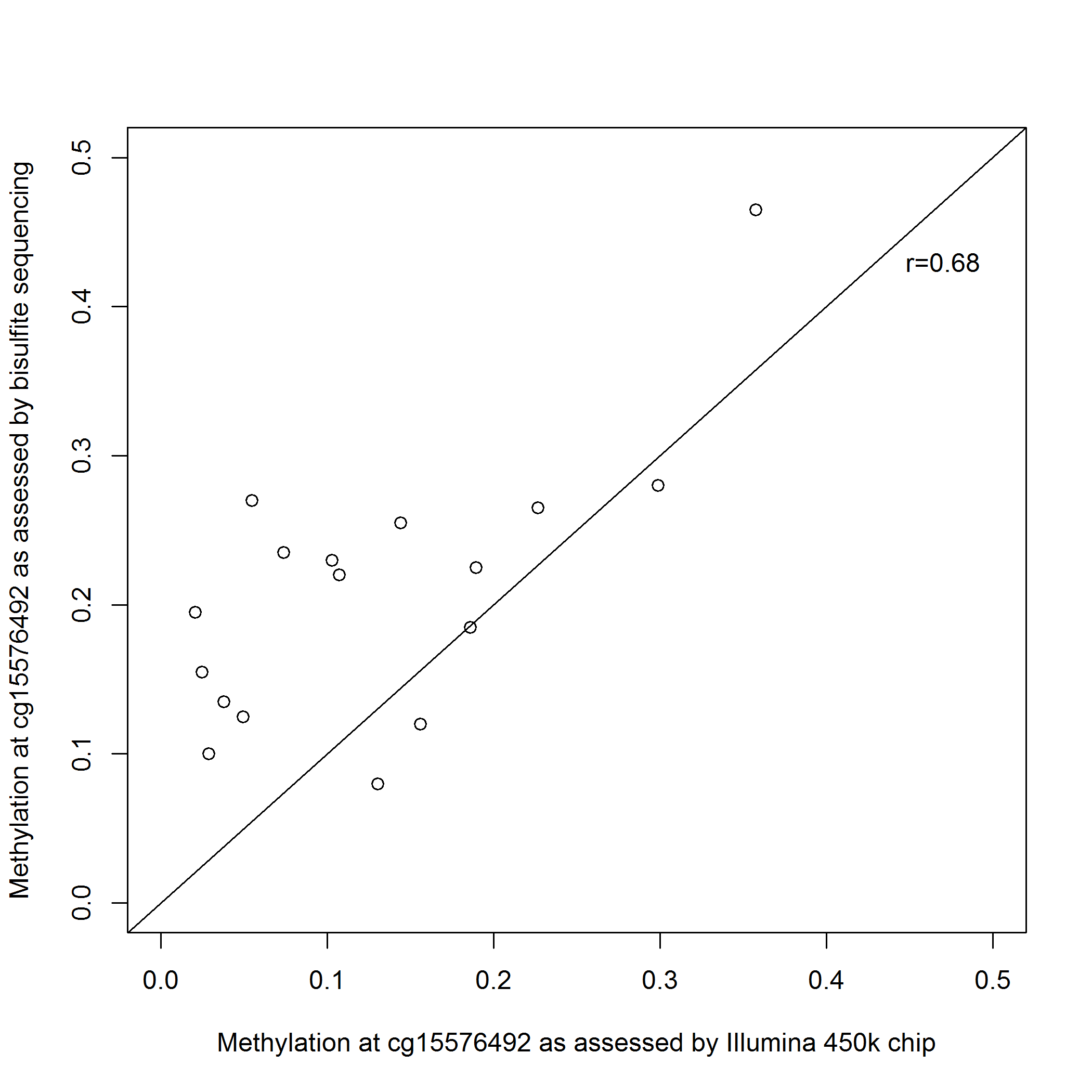

Supplement: Additional file 9: — Correlations between Illumina 450 K and pyrosequence analysis of cg15576492. Methylation at cg15576492, as determined by the Illumina 450 k Chip and expressed as β value, is plotted against methylation at cg15576492, as determined by pyrosequencing and expressed as β value (n = 17). (TIFF 12920 kb) [file 13073_2015_225_MOESM9_ESM.tif]
