# Supplementary material for: Many obesity-associated SNPs strongly associate with DNA methylation changes at proximal promoters and enhancers
Source: Genome Med. 2015 Oct 8;7:103. doi: 10.1186/s13073-015-0225-4 (PMC4599317; doi:10.1186/s13073-015-0225-4)
Supplement: Additional file 3: — Cell lines and target transcription factors of the ChIA-PET libraries. #Cell types are described in ENCODE project [89]. *Target transcription factors and their corresponding antibodies are described in the ENCODE project [90]. (DOCX 11 kb) [file 13073_2015_225_MOESM3_ESM.docx]

| Cell type^#^ | Replicate | Target transcription factor* |
| --- | --- | --- |
| MCF-7 | 1 | ERα |
| MCF-7 | 2 | ERα |
| MCF-7 | 3 | ERα |
| MCF-7 | 1 | RNA Polymerase II |
| MCF-7 | 2 | RNA Polymerase II |
| MCF-7 | 3 | RNA Polymerase II |
| MCF-7 | 4 | RNA Polymerase II |
| MCF-7 | 1 | CTCF |
| MCF-7 | 2 | CTCF |
| K562 | 1 | RNA Polymerase II |
| K562 | 2 | RNA Polymerase II |
| K562 | 1 | CTCF |
| HCT116 | 1 | RNA Polymerase II |
| HeLa | 1 | RNA Polymerase II |
| NB4 | 1 | RNA Polymerase II |
