# Supplementary material for: Many obesity-associated SNPs strongly associate with DNA methylation changes at proximal promoters and enhancers
Source: Genome Med. 2015 Oct 8;7:103. doi: 10.1186/s13073-015-0225-4 (PMC4599317; doi:10.1186/s13073-015-0225-4)
Supplement: Additional file 4: — Description of the 107 associated CpGs found in blood and their annotation. *Coefficient of the linear model associated with the obesity-associated SNP: positive for increased methylation with presence of risk allele; coefficients are calculated using M values. (DOCX 38 kb) [file 13073_2015_225_MOESM4_ESM.docx]

| **Annotation** | | | | **Discovery analysis** | | | | |  |
| --- | --- | --- | --- | --- | --- | --- | --- | --- | --- |
| SNP | CpG | In the promoter of | In a region showing long-range interaction with the promoter of | SNP coefficient in blood (n=355)* | SNP effect size (% of variance explained) | SNP raw p-value | SNP q-value | Weight Category effect size (% of variance explained) | Weight Category raw p-value |
| rs1011731 | cg13446689 |  |  | 0.357 | 14.3 | 7.76e-14 | 3.21e-11 | 0.836 | 0.115 |
| rs10150332 | cg07177395 |  |  | -0.138 | 3.54 | 0.000385 | 0.0318 | 0.0458 | 0.42 |
| rs1055144 | cg00935653 |  | *RNU6-16P, CBX3, HNRNPA2B1* | 0.116 | 3.67 | 0.000621 | 0.0473 | 2.43 | 0.000958 |
| rs1055144 | cg03190219 |  | *CBX3, HNRNPA2B1* | 0.307 | 4.75 | 1.76e-05 | 0.00204 | 2.73 | 0.000875 |
| rs1055144 | cg05149343 |  | *RNU6-16P, CBX3, HNRNPA2B1* | 0.111 | 6.88 | 1.01e-06 | 0.00016 | 0.268 | 0.417 |
| rs1055144 | cg09596116 | *MIR148A* | *CBX3, HNRNPA2B1* | 0.19 | 5.03 | 4.42e-05 | 0.0047 | 0.000215 | 0.931 |
| rs1055144 | cg13710556 |  | *CBX3, HNRNPA2B1, CYCS* | 0.214 | 6.21 | 1.3e-06 | 0.000195 | 0.00181 | 0.976 |
| rs1055144 | cg15575538 |  | *CBX3, HNRNPA2B1, CYCS* | 0.234 | 5.92 | 9.21e-07 | 0.000149 | 1.01 | 0.0443 |
| rs10767664 | cg09781307 |  |  | -0.306 | 7.48 | 1.35e-08 | 3.29e-06 | 0.199 | 0.416 |
| rs10767664 | cg10635145 | *BDNF* |  | 0.322 | 17.7 | 2.4e-17 | 1.33e-14 | 0.504 | 0.074 |
| rs10767664 | cg18117895 | *BDNF* | *METTL15, KIF18A* | -0.0921 | 8.05 | 8.71e-08 | 1.83e-05 | 0.0501 | 0.721 |
| rs10767664 | cg26949694 | *BDNF* |  | 0.137 | 2.42 | 0.000513 | 0.0398 | 0.513 | 0.195 |
| rs10769908 | cg01677628 |  |  | -0.285 | 8.71 | 1.76e-08 | 4.14e-06 | 0.261 | 0.417 |
| rs10769908 | cg07138994 |  |  | -0.261 | 10.2 | 3.22e-10 | 9.64e-08 | 0.434 | 0.273 |
| rs10769908 | cg10639395 |  | *ST5, TRIM66, LOC102724784, AKIP1* | -0.182 | 4.75 | 1.79e-05 | 0.00204 | 0.504 | 0.136 |
| rs10769908 | cg27431761 |  |  | -0.354 | 12.1 | 2.39e-11 | 7.43e-09 | 0.0283 | 0.856 |
| rs10838738 | cg00214780 | *PTPMT1* | *NDUFS3, KBTBD4, CELF1, SLC39A13* | 0.1 | 3.72 | 0.000267 | 0.0228 | 0.0575 | 0.581 |
| rs10838738 | cg04959790 | *NR1H3* | *AFRGAP2, MIR6745, DDB2, ACP2, CELF1, ETFB, LOC101928943, SPI1, PACSIN3* | -0.118 | 4.37 | 2.88e-06 | 4,00E-04 | 1.05 | 0.138 |
| rs10838738 | cg05377527 | *PTPMT1* | *NDUFS3, KBTBD4, CELF1, SLC39A13, C1QTNF4, MTCH2* | 0.126 | 16.8 | 1.44e-15 | 6.6e-13 | 0.00712 | 0.94 |
| rs10838738 | cg05585544 |  | *NDUFS3, KBTBD4, C1QTNF4* | -0.171 | 4.64 | 0.000122 | 0.0114 | 0.582 | 0.151 |
| rs10838738 | cg13308137 |  | *NUP160* | -0.629 | 6.52 | 2.01e-07 | 3.91e-05 | 0.685 | 0.106 |
| rs10838738 | cg14232165 |  | *PTPMT1, NDUFS3, KBTBD4* | 0.17 | 4.78 | 9.87e-05 | 0.00983 | 0.0101 | 0.858 |
| rs10838738 | cg18512352 |  | *NDUFS3, KBTBD4, CELF1* | -0.249 | 7.86 | 7.28e-21 | 5.14e-18 | 0.161 | 0.178 |
| rs10838738 | cg20135002 |  | *FAM180B, C1QTNF4* | -0.252 | 18.2 | 1.31e-16 | 6.34e-14 | 0.193 | 0.335 |
| rs1152846 | cg04441577 |  |  | 0.31 | 8.01 | 3.71e-07 | 6.72e-05 | 0.181 | 0.586 |
| rs12517906 | cg00514575 | *MGAT1* | *ZFP62, HEIH, TRIM7, LINC00847* | 1.18 | 11.8 | 1.26e-09 | 3.5e-07 | 0.0428 | 0.652 |
| rs12517906 | cg13004587 | *SCGB3A1* |  | 0.213 | 4.52 | 1.68e-05 | 0.00198 | 0.752 | 0.105 |
| rs1443512 | cg07731191 |  | *HOXC11, HOTAIR* | 0.342 | 8.16 | 4.57e-07 | 7.9e-05 | 0.0207 | 0.806 |
| rs1443512 | cg16295056 |  | *HOXC9, HOXC-AS1, HOXC-AS2, MIR196A2* | 0.304 | 7.23 | 2.04e-06 | 0.000294 | 0.0442 | 0.734 |
| rs1443512 | cg16305379 | *HOXC12* | *CISTR, HOTAIR, HOXC11* | -0.138 | 5.97 | 7.15e-05 | 0.00727 | 0.00418 | 0.961 |
| rs1443512 | cg25024717 |  |  | 0.145 | 3.87 | 0.000142 | 0.0127 | 0.298 | 0.355 |
| rs17782313 | cg22549408 | *PMAIP1* |  | -0.159 | 2.46 | 0.000408 | 0.033 | 0.224 | 0.281 |
| rs1878047 | cg04588972 |  | *SNORD88B, KLK1, MGC45922, KLK15, SNORD88C, C19orf48* | -0.0582 | 3.64 | 0.000434 | 0.0348 | 0.101 | 0.587 |
| rs1878047 | cg14884932 |  |  | -0.278 | 9.09 | 1.87e-08 | 4.27e-06 | 0.108 | 0.683 |
| rs1878047 | cg15497724 |  |  | -0.337 | 5.71 | 3.53e-06 | 0.000482 | 0.128 | 0.353 |
| rs1927702 | cg01560422 | *PSIP1* | *CCDC171* | 0.156 | 2.62 | 0.000677 | 0.0499 | 0.608 | 0.141 |
| rs206936 | cg23117447 | *RPS10, RPS10-NUDT3* | *HMGA1, MIR6835, C6orf1, NUDT3, SNRPC, UHRF1BP1, BRD2, PACSIN1* | 0.0909 | 4.2 | 7.2e-05 | 0.00727 | 0.547 | 0.164 |
| rs2112347 | cg03649429 |  | *POC5, COL4A3BP, POLK* | 0.551 | 6.1 | 4.36e-06 | 0.000572 | 0.0173 | 0.803 |
| rs2241423 | cg07010088 |  | *SKOR1, MAP2K5* | -0.158 | 3.48 | 0.000168 | 0.0147 | 0.295 | 0.198 |
| rs2241423 | cg09469610 | *SKOR1* | *LOC101929076* | -0.119 | 8.09 | 2.51e-08 | 5.57e-06 | 0.192 | 0.293 |
| rs2241423 | cg09917562 |  | *LOC101929076* | 0.429 | 36.6 | 1.48e-36 | 2.3e-33 | 0.114 | 0.514 |
| rs2241423 | cg24579218 |  |  | 0.695 | 56.8 | 6.07e-68 | 4.72e-64 | 0.166 | 0.233 |
| rs2241423 | cg26545918 |  | *IQCH-AS1, LOC101929076, MAP2K5, C15orf61* | -0.135 | 5.54 | 3.33e-08 | 7.2e-06 | 0.0637 | 0.307 |
| rs2241423 | cg27219399 | *MAP2K5* | *IQCH-AS1, C15orf61, LOC101929076* | -0.233 | 12 | 3.37e-12 | 1.19e-09 | 0.00189 | 0.693 |
| rs2287019 | cg02473103 | *SIX5* | *QPCTL, SNRPD2, FBXO46, LOC388553, SYMPK, FOXA3, EML2, MIR330, C19orf83, GIPR, IRF2BP1, DMWD, FOSB, RSPH6A* | 0.0619 | 3.47 | 0.000396 | 0.0324 | 0.000569 | 0.939 |
| rs2287019 | cg04282912 |  | *RSPH6A* | -0.482 | 3.67 | 0.000331 | 0.0279 | 0.775 | 0.0953 |
| rs2287019 | cg13320842 |  | *EML2, MIR330, C19orf83, GIPR, VASP, QPCTL, SNRPD2, FBXO46, SIX5, MIR642B, MIR642A* | -0.38 | 7.59 | 2.8e-07 | 5.31e-05 | 0.0365 | 0.901 |
| rs2287019 | cg18735402 |  | *MIR642B, DMPK, GIPR, MIR642A, QPCTL, SNRPD2, SIX5, PPP1R13L, CD3EAP, EML2, IRF2BP1, OPA3, C19orf183* | 0.499 | 12.2 | 8.99e-14 | 3.49e-11 | 0.409 | 0.164 |
| rs2287019 | cg19822309 |  | *MIR642B, SNRPD2, DMPK, GIPR, MIR642A, QPCTL, SIX5, PPP1R13L, CD3EAP, EML2, IRF2BP1, OPA3, C19orf183* | 0.415 | 22 | 1.69e-26 | 1.64e-23 | 0.495 | 0.0591 |
| rs2287019 | cg20434926 |  | *MIR642B, DMPK, GIPR, MIR642A, QPCTL, SNRPD2, SIX5, PPP1R13L, CD3EAP, EML2, IRF2BP1, OPA3, C19orf183* | 0.304 | 5.91 | 2.51e-06 | 0.000355 | 0.892 | 0.0537 |
| rs2444217 | cg00834536 |  |  | 0.105 | 3.46 | 5.2e-05 | 0.00539 | 0.462 | 0.249 |
| rs2444217 | cg07628416 |  | *LOC100507501, GLIS2, NLRC3, ADCY9, TFAP4* | -0.223 | 7.75 | 3.91e-07 | 6.91e-05 | 0.00118 | 0.849 |
| rs2444217 | cg08098950 |  | *LOC100507501, GLIS2, NLRC3, TFAP4, ADCY9, LOC102724927* | -0.201 | 8.37 | 1.51e-07 | 3,00E-05 | 2.45e-05 | 0.976 |
| rs2444217 | cg09300795 |  |  | 0.218 | 12.2 | 5.6e-17 | 2.9e-14 | 0.378 | 0.0742 |
| rs2815752 | cg09256413 |  |  | -0.168 | 5.66 | 1.33e-05 | 0.00164 | 0.152 | 0.294 |
| rs3934834 | cg00305285 |  |  | 0.0922 | 4.92 | 3.29e-05 | 0.0036 | 0.324 | 0.269 |
| rs3934834 | cg02105666 |  | *HES4, AGRN, ISG15, PUSL1, ACAP3, SAMD11, KLHL17, NOC2L, C1orf159, PERM1, MIR200B, UBE2J2* | -0.181 | 6.48 | 9.2e-07 | 0.000149 | 0.293 | 0.307 |
| rs3934834 | cg02341264 |  | *AGRN, TTLL10* | -0.241 | 2.81 | 0.000647 | 0.0484 | 0.0869 | 0.646 |
| rs3934834 | cg07549208 |  | *HES4, AGRN, PUSL1, ACAP3, KLHL17, NOC2L, ISG15, C1orf159, PERM1, MIR200B, UBE2J2* | -0.285 | 6.05 | 4.26e-06 | 0.000571 | 0.593 | 0.125 |
| rs3934834 | cg07787977 |  | *HES4, RNF223* | 0.091 | 3.83 | 0.00044 | 0.0349 | 0.000478 | 0.781 |
| rs3934834 | cg09363892 | *AGRN* | *KLHL17, NOC2L, PERM1, UBE2J2, HES4, ISG15, SCNN1D, C1orf159, B3GALT6, SDF4, LOC100130417, RNF223* | -0.176 | 3.02 | 0.000373 | 0.0312 | 0.104 | 0.77 |
| rs3934834 | cg11200797 |  | *RNF223, HES4, ISG15, AGRN, C1orf159, PUSL1, ACAP3, MIR200B, B3GALT6, SDF4, FAM132A, UBE2J2, MIR6726* | -0.682 | 10.7 | 4.25e-10 | 1.22e-07 | 0.279 | 0.276 |
| rs3934834 | cg15500259 |  | *HES4, AGRN, ISG15, C1orf159, CPSF3L, PLEKHN1, PERM1, UBE2J2, CPTP* | 0.156 | 4.6 | 3.09e-05 | 0.00343 | 0.0802 | 0.377 |
| rs3934834 | cg15576492 |  | *PERM1, HES4, ISG15, RNF223, AGRN, C1orf159, PUSL1, ACAP3, MIR200B, B3GALT6, SDF4, FAM132A, UBE2J2, MIR6726* | -1.53 | 34.2 | 2.39e-33 | 3.1e-30 | 0.113 | 0.575 |
| rs3934834 | cg17021880 |  | *UBE2J2, RNF223* | 0.576 | 11.7 | 6.77e-12 | 2.29e-09 | 0.281 | 0.369 |
| rs3934834 | cg18432292 |  | *HES4, UB2J2, AGRN, KLHL17, NOC2L, PLEKHN1, PERM1, ISG15, C1orf159, CPSF3L, CPTP, MIR200B, MIR200A, B3GALT6, SDF4, FAM132A, PUSL1, ACAP3* | -0.145 | 5.06 | 6.25e-06 | 0.000783 | 1.22 | 0.0358 |
| rs3934834 | cg20685419 |  | *PLEKHN1, HES4, KLHL17, NOC2L, ISG15, C1orf159, LINC01342, UBE2J2, PUSL1, ACAP3, PERM1, AGRN, MIR200B, MIR200A, B3GALT6, SDF4* | -0.0848 | 5.29 | 4.42e-06 | 0.000572 | 0.33 | 0.0924 |
| rs3934834 | cg21139076 |  | *HES4, AGRN, PUSL1, ACAP3, KLHL17, NOC2L, ISG15, C1orf159, PERM1, MIR200B, UBE2J2* | -0.262 | 8.84 | 1.25e-08 | 3.14e-06 | 1.27 | 0.0299 |
| rs3934834 | cg22044028 |  | *RNF223, PERM1, HES4, ISG15, AGRN, C1orf159, PUSL1, ACAP3, MIR200B, B3GALT6, SDF4, FAM132A, UBE2J2, MIR6726* | -1.28 | 29.2 | 8.13e-28 | 9.03e-25 | 0.199 | 0.429 |
| rs3934834 | cg22864340 |  | *TTLL10, B3GALT6, SDF4, AGRN, RNF223, TNFRSF18, UBE2J2* | -0.263 | 4.3 | 0.000447 | 0.0351 | 0.0251 | 0.737 |
| rs652722 | cg11385473 | *IMMP1L, ELP4* |  | 0.201 | 2.39 | 0.000643 | 0.0484 | 0.134 | 0.444 |
| rs6784615 | cg04865290 |  | *TMEM110-MUSTN1, TMEM110* | 0.996 | 10.1 | 1.08e-11 | 3.48e-09 | 0.579 | 0.13 |
| rs6784615 | cg07615364 |  | *RRP9, PARP3, RPL29, LINC00696, POC1A, ACY1, ALAS1* | 0.208 | 1.38 | 0.000686 | 0.0499 | 0.308 | 0.178 |
| rs6784615 | cg11645453 | *ITIH4* |  | 0.498 | 3.51 | 0.000121 | 0.0114 | 1.13 | 0.0581 |
| rs6784615 | cg16362603 | *SEMA3G* | *WDR82* | 0.315 | 7.87 | 6.89e-07 | 0.000116 | 1.9e-06 | 0.749 |
| rs6784615 | cg18404041 |  |  | -0.247 | 4.14 | 0.000127 | 0.0116 | 1.06 | 0.112 |
| rs6795735 | cg23078228 |  |  | -0.16 | 5.59 | 4,00E-05 | 0.00432 | 0.619 | 0.139 |
| rs6861681 | cg06889108 |  |  | -0.117 | 2.78 | 2.85e-05 | 0.00321 | 0.0129 | 0.819 |
| rs6861681 | cg18693985 |  |  | -1.13 | 39.5 | 1.93e-43 | 4.99e-40 | 0.456 | 0.118 |
| rs6861681 | cg18757087 |  |  | -0.156 | 4.5 | 3.69e-07 | 6.72e-05 | 0.0888 | 0.715 |
| rs6861681 | cg21566177 |  | *BOD1* | -0.53 | 15.6 | 1.08e-20 | 6.98e-18 | 0.762 | 0.0117 |
| rs713586 | cg01884057 |  |  | 0.752 | 53.8 | 7.66e-64 | 2.98e-60 | 0.0219 | 0.729 |
| rs713586 | cg09505516 |  |  | -0.38 | 19.4 | 1.51e-19 | 9.02e-17 | 1.5 | 0.00966 |
| rs713586 | cg11023668 |  |  | -0.636 | 37.3 | 3.41e-43 | 6.62e-40 | 0.346 | 0.0955 |
| rs713586 | cg16302441 | *POMC* |  | -0.147 | 4.98 | 0.000105 | 0.0103 | 0.0249 | 0.861 |
| rs713586 | cg16888658 | *ADCY3* | *DNAJC27, DNAJC27-AS1, EFR3B* | -0.166 | 3.42 | 0.000116 | 0.0113 | 0.000612 | 0.897 |
| rs713586 | cg23809645 |  | *POMC* | 0.208 | 3.44 | 0.000215 | 0.0186 | 0.379 | 0.259 |
| rs713586 | cg26038461 |  |  | -0.111 | 5.41 | 1.18e-06 | 0.000184 | 0.242 | 0.265 |
| rs713586 | cg27107076 |  | *CENPO, PTRHD1* | -0.174 | 6.94 | 1.1e-07 | 2.25e-05 | 0.963 | 0.035 |
| rs718314 | cg02058108 | *SSPN* |  | 0.174 | 4.33 | 4.76e-05 | 0.00501 | 0.233 | 0.145 |
| rs7481311 | cg06731443 | *LGR4* | *CCDC34* | 0.262 | 3.41 | 0.00012 | 0.0114 | 0.0375 | 0.616 |
| rs7481311 | cg14346046 | *LGR4* | *CCDC34* | 0.215 | 4.81 | 1.46e-05 | 0.00174 | 0.102 | 0.527 |
| rs7481311 | cg18117895 | *BDNF* |  | -0.0537 | 3.36 | 0.000526 | 0.0405 | 0.127 | 0.528 |
| rs7498665 | cg00201760 | *IL27* | *CCDC101* | -0.0697 | 0.408 | 0.000684 | 0.0499 | 0.0195 | 0.914 |
| rs7498665 | cg00348858 | *TUFM* | *MIR4721, RABEP2, NFATC2IP, ATP2A1, LOC100289092, MIR4517, SH2B1* | -0.155 | 5.78 | 1.21e-06 | 0.000185 | 0.409 | 0.265 |
| rs7498665 | cg00489954 | *MIR4721* | *TUFM, SH2B1* | 0.138 | 8.69 | 5.49e-09 | 1.42e-06 | 0.0641 | 0.387 |
| rs7498665 | cg01378222 | *SULT1A1* | *CCDC101* | 0.182 | 5.53 | 1.41e-05 | 0.00172 | 0.166 | 0.221 |
| rs7498665 | cg01621080 | *SULT1A2* | *CCDC101* | -0.17 | 8.78 | 2.31e-09 | 6.18e-07 | 0.267 | 0.204 |
| rs7498665 | cg03300649 | *APOBR, CLN3* | *CCDC101, IL27* | -0.0941 | 4.28 | 0.000125 | 0.0116 | 0.00059 | 0.915 |
| rs7498665 | cg04270652 | *SULT1A2* | *CCDC101* | -0.367 | 22.1 | 1.21e-21 | 1.05e-18 | 0.00134 | 0.692 |
| rs7498665 | cg08180572 | *SPNS1* | *ATXN2L, NFATC2IP, SH2B1, RABEP2* | -0.146 | 4.1 | 0.000129 | 0.0117 | 0.187 | 0.487 |
| rs7498665 | cg08761264 | *SH2B1* | *TUFM, ATXN2L, MIR4721, ATP2A1, LOC100289092, RABEP2, NFATC2IP, CD19, SPNS1* | -0.121 | 5.91 | 6.14e-06 | 0.000783 | 0.0916 | 0.663 |
| rs7498665 | cg09754948 | *ATXN2L* | *CLDN6, TNFRSF12A, SH2B1, ATP2A1, LOC100289092, NFATC2IP, RABEP2, SPNS1, TUFM, SBK1, CCDC101, CD19* | 0.238 | 22.6 | 3.45e-21 | 2.68e-18 | 0.000674 | 0.899 |
| rs7498665 | cg26792089 | *IL27* | *CCDC101* | -0.0819 | 2.74 | 1.46e-06 | 0.000214 | 0.252 | 0.136 |
| rs7498665 | cg27413008 | *IL27* | *CLN3* | -0.363 | 12.7 | 7.84e-14 | 3.21e-11 | 0.000532 | 0.705 |
| rs984222 | cg07961512 |  |  | 0.378 | 14 | 1.25e-13 | 4.64e-11 | 0.458 | 0.241 |
| rs984222 | cg17592360 |  | *WARS2* | 0.153 | 4.07 | 0.000149 | 0.0132 | 0.0162 | 0.79 |
