# Supplementary material for: Many obesity-associated SNPs strongly associate with DNA methylation changes at proximal promoters and enhancers
Source: Genome Med. 2015 Oct 8;7:103. doi: 10.1186/s13073-015-0225-4 (PMC4599317; doi:10.1186/s13073-015-0225-4)
Supplement: Additional file 6: — Chromatin states at the genomic position of the 107 CpGs, in the 11 investigated tissues. AN adipose nuclei, BrainAC brain anterior caudate, BrainAG brain angular gyrus, BrainCG brain cingulate gyrus, BrainHIPPO brain hippocampus, BrainITL brain inferior temporal lobe, BrainSN brain substantia nigra, PBMC peripheral blood mononuclear primary cells, PI pancreatic islets, SM skeletal muscle. (DOCX 36 kb) [file 13073_2015_225_MOESM6_ESM.docx]

| **SNP** | **CpG** | **AN** | **BrainAC** | **BrainAG** | **BrainCG** | **BrainHIPPO** | **BrainITL** | **BrainSN** | **Liver** | **PBMC** | **PI** | **SM** |
| --- | --- | --- | --- | --- | --- | --- | --- | --- | --- | --- | --- | --- |
| rs1011731 | cg13446689 | Promoter-associated | Enhancer | Enhancer | Promoter-associated | Enhancer | Enhancer | Enhancer | Enhancer | Enhancer | Enhancer | Enhancer |
| rs10150332 | cg07177395 | Quiescent | Quiescent | Enhancer | Quiescent | Quiescent | Quiescent | Enhancer | Quiescent | Quiescent | Quiescent | Quiescent |
| rs1055144 | cg00935653 | Polycomb-repressed | Polycomb-repressed | Polycomb-repressed | Polycomb-repressed | Polycomb-repressed | Polycomb-repressed | Polycomb-repressed | Polycomb-repressed | Polycomb-repressed | Polycomb-repressed | Promoter-associated |
| rs1055144 | cg03190219 | Polycomb-repressed | Polycomb-repressed | Polycomb-repressed | Polycomb-repressed | Polycomb-repressed | Polycomb-repressed | Polycomb-repressed | Polycomb-repressed | Polycomb-repressed | Polycomb-repressed | Polycomb-repressed |
| rs1055144 | cg05149343 | Promoter-associated | Polycomb-repressed | Polycomb-repressed | Polycomb-repressed | Polycomb-repressed | Promoter-associated | Polycomb-repressed | Polycomb-repressed | Polycomb-repressed | Polycomb-repressed | Promoter-associated |
| rs1055144 | cg09596116 | Promoter-associated | Promoter-associated | Promoter-associated | Promoter-associated | Promoter-associated | Promoter-associated | Promoter-associated | Promoter-associated | Promoter-associated | Promoter-associated | Promoter-associated |
| rs1055144 | cg13710556 | Enhancer | Polycomb-repressed | Polycomb-repressed | Polycomb-repressed | Polycomb-repressed | Polycomb-repressed | Polycomb-repressed | Polycomb-repressed | Polycomb-repressed | Polycomb-repressed | Enhancer |
| rs1055144 | cg15575538 | Enhancer | Polycomb-repressed | Polycomb-repressed | Polycomb-repressed | Polycomb-repressed | Polycomb-repressed | Polycomb-repressed | Polycomb-repressed | Polycomb-repressed | Polycomb-repressed | Enhancer |
| rs10767664 | cg09781307 | Quiescent | Polycomb-repressed | Quiescent | Quiescent | Quiescent | Quiescent | Quiescent | Quiescent | Quiescent | Quiescent | Quiescent |
| rs10767664 | cg10635145 | Polycomb-repressed | Polycomb-repressed | Polycomb-repressed | Polycomb-repressed | Polycomb-repressed | Polycomb-repressed | Polycomb-repressed | Polycomb-repressed | Polycomb-repressed | Promoter-associated | Polycomb-repressed |
| rs10767664 | cg18117895 | Promoter-associated | Promoter-associated | Promoter-associated | Promoter-associated | Promoter-associated | Promoter-associated | Promoter-associated | Promoter-associated | Promoter-associated | Promoter-associated | Promoter-associated |
| rs10767664 | cg26949694 | Polycomb-repressed | Polycomb-repressed | Polycomb-repressed | Polycomb-repressed | Polycomb-repressed | Promoter-associated | Polycomb-repressed | Polycomb-repressed | Polycomb-repressed | Polycomb-repressed | Polycomb-repressed |
| rs10769908 | cg01677628 | Polycomb-repressed | Quiescent | Polycomb-repressed | Polycomb-repressed | Polycomb-repressed | Quiescent | Quiescent | Polycomb-repressed | Quiescent | Quiescent | Polycomb-repressed |
| rs10769908 | cg07138994 | Polycomb-repressed | Quiescent | Polycomb-repressed | Polycomb-repressed | Polycomb-repressed | Quiescent | Quiescent | Polycomb-repressed | Quiescent | Quiescent | Polycomb-repressed |
| rs10769908 | cg10639395 | Promoter-associated | Promoter-associated | Promoter-associated | Promoter-associated | Promoter-associated | Promoter-associated | Promoter-associated | Promoter-associated | Promoter-associated | Promoter-associated | Promoter-associated |
| rs10769908 | cg27431761 | Polycomb-repressed | Quiescent | Polycomb-repressed | Polycomb-repressed | Polycomb-repressed | Quiescent | Quiescent | Polycomb-repressed | Quiescent | Quiescent | Polycomb-repressed |
| rs10838738 | cg00214780 | Quiescent | Quiescent | Quiescent | Quiescent | Quiescent | Quiescent | Quiescent | Enhancer | Enhancer | Enhancer | Quiescent |
| rs10838738 | cg04959790 | Enhancer | Enhancer | Enhancer | Enhancer | Enhancer | Enhancer | Promoter-associated | Enhancer | Enhancer | Quiescent | Enhancer |
| rs10838738 | cg05377527 | Promoter-associated | Promoter-associated | Promoter-associated | Promoter-associated | Promoter-associated | Promoter-associated | Promoter-associated | Promoter-associated | Promoter-associated | Promoter-associated | Promoter-associated |
| rs10838738 | cg05585544 | Active transcription | Enhancer | Quiescent | Quiescent | Quiescent | Quiescent | Quiescent | Quiescent | Quiescent | Quiescent | Quiescent |
| rs10838738 | cg13308137 | Enhancer | Enhancer | Enhancer | Enhancer | Enhancer | Enhancer | Enhancer | Enhancer | Enhancer | Enhancer | Enhancer |
| rs10838738 | cg14232165 | Enhancer | Enhancer | Enhancer | Enhancer | Enhancer | Quiescent | Quiescent | Enhancer | Enhancer | Enhancer | Enhancer |
| rs10838738 | cg18512352 | Active transcription | Enhancer | Enhancer | Enhancer | Active transcription | Enhancer | Enhancer | Active transcription | Enhancer | Active transcription | Enhancer |
| rs10838738 | cg20135002 | Enhancer | Enhancer | Enhancer | Enhancer | Enhancer | Enhancer | Enhancer | Active transcription | Quiescent | Active transcription | Enhancer |
| rs1152846 | cg04441577 | Enhancer | Enhancer | Quiescent | Enhancer | Enhancer | Quiescent | Enhancer | Enhancer | Enhancer | Enhancer | Enhancer |
| rs12517906 | cg00514575 | Promoter-associated | Enhancer | Enhancer | Promoter-associated | Enhancer | Enhancer | Enhancer | Enhancer | Promoter-associated | Enhancer | Enhancer |
| rs12517906 | cg13004587 | Polycomb-repressed | Promoter-associated | Polycomb-repressed | Polycomb-repressed | Polycomb-repressed | Polycomb-repressed | Polycomb-repressed | Quiescent | Promoter-associated | Polycomb-repressed | Polycomb-repressed |
| rs1443512 | cg07731191 | Polycomb-repressed | Polycomb-repressed | Polycomb-repressed | Polycomb-repressed | Polycomb-repressed | Polycomb-repressed | Polycomb-repressed | Polycomb-repressed | Polycomb-repressed | Polycomb-repressed | Polycomb-repressed |
| rs1443512 | cg16295056 | Polycomb-repressed | Polycomb-repressed | Polycomb-repressed | Polycomb-repressed | Polycomb-repressed | Polycomb-repressed | Polycomb-repressed | Polycomb-repressed | Polycomb-repressed | Polycomb-repressed | Promoter-associated |
| rs1443512 | cg16305379 | Polycomb-repressed | Polycomb-repressed | Polycomb-repressed | Polycomb-repressed | Polycomb-repressed | Polycomb-repressed | Polycomb-repressed | Polycomb-repressed | Polycomb-repressed | Polycomb-repressed | Polycomb-repressed |
| rs1443512 | cg25024717 | Polycomb-repressed | Polycomb-repressed | Polycomb-repressed | Polycomb-repressed | Polycomb-repressed | Polycomb-repressed | Polycomb-repressed | Polycomb-repressed | Polycomb-repressed | Polycomb-repressed | Polycomb-repressed |
| rs17782313 | cg22549408 | Promoter-associated | Promoter-associated | Promoter-associated | Polycomb-repressed | Promoter-associated | Promoter-associated | Polycomb-repressed | Promoter-associated | Promoter-associated | Promoter-associated | Promoter-associated |
| rs1878047 | cg04588972 | Promoter-associated | Promoter-associated | Promoter-associated | Promoter-associated | Promoter-associated | Promoter-associated | Promoter-associated | Promoter-associated | Promoter-associated | Promoter-associated | Polycomb-repressed |
| rs1878047 | cg14884932 | Polycomb-repressed | Polycomb-repressed | Polycomb-repressed | Polycomb-repressed | Polycomb-repressed | Polycomb-repressed | Polycomb-repressed | Polycomb-repressed | Quiescent | Polycomb-repressed | Polycomb-repressed |
| rs1878047 | cg15497724 | Polycomb-repressed | Polycomb-repressed | Polycomb-repressed | Polycomb-repressed | Polycomb-repressed | Polycomb-repressed | Polycomb-repressed | Polycomb-repressed | Quiescent | Polycomb-repressed | Polycomb-repressed |
| rs1927702 | cg01560422 | Active transcription | Promoter-associated | Promoter-associated | Promoter-associated | Promoter-associated | Promoter-associated | Enhancer | Active transcription | Enhancer | Active transcription | Promoter-associated |
| rs206936 | cg23117447 | Promoter-associated | Promoter-associated | Promoter-associated | Promoter-associated | Promoter-associated | Promoter-associated | Promoter-associated | Promoter-associated | Promoter-associated | Promoter-associated | Promoter-associated |
| rs2112347 | cg03649429 | Active transcription | Active transcription | Quiescent | Quiescent | Quiescent | Quiescent | Quiescent | Active transcription | Quiescent | Active transcription | Quiescent |
| rs2241423 | cg07010088 | Polycomb-repressed | Polycomb-repressed | Polycomb-repressed | Polycomb-repressed | Polycomb-repressed | Polycomb-repressed | Polycomb-repressed | Polycomb-repressed | Promoter-associated | Promoter-associated | Polycomb-repressed |
| rs2241423 | cg09469610 | Polycomb-repressed | Promoter-associated | Polycomb-repressed | Polycomb-repressed | Promoter-associated | Polycomb-repressed | Polycomb-repressed | Promoter-associated | Promoter-associated | Polycomb-repressed | Polycomb-repressed |
| rs2241423 | cg09917562 | Polycomb-repressed | Polycomb-repressed | Polycomb-repressed | Polycomb-repressed | Promoter-associated | Polycomb-repressed | Polycomb-repressed | Polycomb-repressed | Polycomb-repressed | Promoter-associated | Polycomb-repressed |
| rs2241423 | cg24579218 | Polycomb-repressed | Quiescent | Quiescent | Quiescent | Quiescent | Quiescent | Quiescent | Quiescent | Quiescent | Quiescent | Polycomb-repressed |
| rs2241423 | cg26545918 | Polycomb-repressed | Polycomb-repressed | Polycomb-repressed | Polycomb-repressed | Polycomb-repressed | Polycomb-repressed | Polycomb-repressed | Polycomb-repressed | Polycomb-repressed | Polycomb-repressed | Polycomb-repressed |
| rs2241423 | cg27219399 | Promoter-associated | Promoter-associated | Promoter-associated | Promoter-associated | Promoter-associated | Promoter-associated | Promoter-associated | Promoter-associated | Enhancer | Promoter-associated | Promoter-associated |
| rs2287019 | cg02473103 | Promoter-associated | Promoter-associated | Promoter-associated | Promoter-associated | Promoter-associated | Promoter-associated | Enhancer | Enhancer | Promoter-associated | Promoter-associated | Promoter-associated |
| rs2287019 | cg04282912 | Active transcription | Active transcription | Active transcription | Active transcription | Active transcription | Active transcription | Active transcription | Active transcription | Active transcription | Active transcription | Active transcription |
| rs2287019 | cg13320842 | Polycomb-repressed | Polycomb-repressed | Polycomb-repressed | Polycomb-repressed | Polycomb-repressed | Polycomb-repressed | Quiescent | Polycomb-repressed | Polycomb-repressed | Enhancer | Polycomb-repressed |
| rs2287019 | cg18735402 | Polycomb-repressed | Promoter-associated | Promoter-associated | Promoter-associated | Promoter-associated | Promoter-associated | Promoter-associated | Promoter-associated | Promoter-associated | Enhancer | Promoter-associated |
| rs2287019 | cg19822309 | Polycomb-repressed | Promoter-associated | Promoter-associated | Promoter-associated | Promoter-associated | Promoter-associated | Promoter-associated | Promoter-associated | Polycomb-repressed | Enhancer | Promoter-associated |
| rs2287019 | cg20434926 | Polycomb-repressed | Promoter-associated | Promoter-associated | Promoter-associated | Promoter-associated | Promoter-associated | Promoter-associated | Promoter-associated | Promoter-associated | Enhancer | Promoter-associated |
| rs2444217 | cg00834536 | Enhancer | Enhancer | Active transcription | Enhancer | Enhancer | Enhancer | Enhancer | Active transcription | Enhancer | Enhancer | Enhancer |
| rs2444217 | cg07628416 | Active transcription | Active transcription | Enhancer | Enhancer | Enhancer | Enhancer | Enhancer | Enhancer | Enhancer | Enhancer | Enhancer |
| rs2444217 | cg08098950 | Active transcription | Active transcription | Enhancer | Enhancer | Enhancer | Enhancer | Enhancer | Enhancer | Enhancer | Enhancer | Enhancer |
| rs2444217 | cg09300795 | Quiescent | Quiescent | Quiescent | Active transcription | Active transcription | Quiescent | Quiescent | Quiescent | Active transcription | Quiescent | Active transcription |
| rs2815752 | cg09256413 | Quiescent | Quiescent | Enhancer | Quiescent | Quiescent | Quiescent | Quiescent | Quiescent | Quiescent | Quiescent | Quiescent |
| rs3934834 | cg00305285 | Quiescent | Quiescent | Quiescent | Quiescent | Quiescent | Quiescent | Quiescent | Quiescent | Quiescent | Quiescent | Quiescent |
| rs3934834 | cg02105666 | Polycomb-repressed | Enhancer | Enhancer | Promoter-associated | Promoter-associated | Promoter-associated | Polycomb-repressed | Enhancer | Enhancer | Enhancer | Polycomb-repressed |
| rs3934834 | cg02341264 | Polycomb-repressed | Enhancer | Enhancer | Enhancer | Enhancer | Enhancer | Enhancer | Enhancer | Promoter-associated | Promoter-associated | Polycomb-repressed |
| rs3934834 | cg07549208 | Polycomb-repressed | Quiescent | Quiescent | Polycomb-repressed | Promoter-associated | Polycomb-repressed | Polycomb-repressed | Quiescent | Enhancer | Enhancer | Polycomb-repressed |
| rs3934834 | cg07787977 | Polycomb-repressed | Quiescent | Enhancer | Quiescent | Enhancer | Enhancer | Enhancer | Quiescent | Polycomb-repressed | Quiescent | Polycomb-repressed |
| rs3934834 | cg09363892 | Promoter-associated | Promoter-associated | Enhancer | Promoter-associated | Enhancer | Enhancer | Promoter-associated | Enhancer | Promoter-associated | Promoter-associated | Promoter-associated |
| rs3934834 | cg11200797 | Quiescent | Quiescent | Quiescent | Quiescent | Quiescent | Quiescent | Quiescent | Quiescent | Quiescent | Quiescent | Polycomb-repressed |
| rs3934834 | cg15500259 | Polycomb-repressed | Quiescent | Quiescent | Polycomb-repressed | Polycomb-repressed | Polycomb-repressed | Polycomb-repressed | Quiescent | Quiescent | Quiescent | Polycomb-repressed |
| rs3934834 | cg15576492 | Quiescent | Enhancer | Enhancer | Promoter-associated | Promoter-associated | Promoter-associated | Enhancer | Enhancer | Promoter-associated | Enhancer | Promoter-associated |
| rs3934834 | cg17021880 | Polycomb-repressed | Quiescent | Quiescent | Quiescent | Quiescent | Quiescent | Quiescent | Quiescent | Polycomb-repressed | Quiescent | Polycomb-repressed |
| rs3934834 | cg18432292 | Polycomb-repressed | Quiescent | Enhancer | Polycomb-repressed | Promoter-associated | Polycomb-repressed | Polycomb-repressed | Enhancer | Promoter-associated | Promoter-associated | Polycomb-repressed |
| rs3934834 | cg20685419 | Polycomb-repressed | Polycomb-repressed | Polycomb-repressed | Polycomb-repressed | Polycomb-repressed | Polycomb-repressed | Polycomb-repressed | Quiescent | Polycomb-repressed | Polycomb-repressed | Polycomb-repressed |
| rs3934834 | cg21139076 | Polycomb-repressed | Quiescent | Quiescent | Polycomb-repressed | Promoter-associated | Polycomb-repressed | Polycomb-repressed | Quiescent | Enhancer | Enhancer | Polycomb-repressed |
| rs3934834 | cg22044028 | Promoter-associated | Promoter-associated | Promoter-associated | Promoter-associated | Promoter-associated | Promoter-associated | Enhancer | Enhancer | Promoter-associated | Enhancer | Promoter-associated |
| rs3934834 | cg22864340 | Polycomb-repressed | Quiescent | Polycomb-repressed | Polycomb-repressed | Polycomb-repressed | Quiescent | Quiescent | Polycomb-repressed | Quiescent | Enhancer | Polycomb-repressed |
| rs652722 | cg11385473 | Promoter-associated | Promoter-associated | Promoter-associated | Promoter-associated | Promoter-associated | Promoter-associated | Promoter-associated | Promoter-associated | Promoter-associated | Promoter-associated | Promoter-associated |
| rs6784615 | cg04865290 | Enhancer | Enhancer | Enhancer | Enhancer | Enhancer | Enhancer | Enhancer | Enhancer | Enhancer | Enhancer | Enhancer |
| rs6784615 | cg07615364 | Promoter-associated | Promoter-associated | Promoter-associated | Promoter-associated | Promoter-associated | Promoter-associated | Promoter-associated | Enhancer | Promoter-associated | Promoter-associated | Promoter-associated |
| rs6784615 | cg11645453 | Polycomb-repressed | Active transcription | Active transcription | Enhancer | Active transcription | Active transcription | Active transcription | Promoter-associated | Active transcription | Enhancer | Enhancer |
| rs6784615 | cg16362603 | Promoter-associated | Promoter-associated | Quiescent | Enhancer | Enhancer | Quiescent | Quiescent | Quiescent | Enhancer | Promoter-associated | Enhancer |
| rs6784615 | cg18404041 | Polycomb-repressed | Polycomb-repressed | Polycomb-repressed | Polycomb-repressed | Quiescent | Polycomb-repressed | Quiescent | Enhancer | Quiescent | Enhancer | Polycomb-repressed |
| rs6795735 | cg23078228 | Quiescent | Polycomb-repressed | Enhancer | Polycomb-repressed | Quiescent | Quiescent | Quiescent | Quiescent | Quiescent | Quiescent | Quiescent |
| rs6861681 | cg06889108 | Promoter-associated | Promoter-associated | Promoter-associated | Promoter-associated | Promoter-associated | Promoter-associated | Promoter-associated | Promoter-associated | Promoter-associated | Promoter-associated | Promoter-associated |
| rs6861681 | cg18693985 | Enhancer | Enhancer | Enhancer | Enhancer | Enhancer | Enhancer | Enhancer | Enhancer | Enhancer | Enhancer | Enhancer |
| rs6861681 | cg18757087 | Promoter-associated | Promoter-associated | Promoter-associated | Promoter-associated | Promoter-associated | Promoter-associated | Promoter-associated | Promoter-associated | Promoter-associated | Promoter-associated | Promoter-associated |
| rs6861681 | cg21566177 | Enhancer | Enhancer | Quiescent | Quiescent | Enhancer | Quiescent | Quiescent | Enhancer | Enhancer | Quiescent | Quiescent |
| rs713586 | cg01884057 | Promoter-associated | Enhancer | Enhancer | Enhancer | Enhancer | Promoter-associated | Promoter-associated | Quiescent | Quiescent | Quiescent | Promoter-associated |
| rs713586 | cg09505516 | Quiescent | Enhancer | Enhancer | Enhancer | Enhancer | Enhancer | Quiescent | Quiescent | Enhancer | Quiescent | Quiescent |
| rs713586 | cg11023668 | Enhancer | Enhancer | Enhancer | Enhancer | Enhancer | Enhancer | Enhancer | Enhancer | Active transcription | Enhancer | Enhancer |
| rs713586 | cg16302441 | Polycomb-repressed | Polycomb-repressed | Polycomb-repressed | Polycomb-repressed | Polycomb-repressed | Polycomb-repressed | Polycomb-repressed | Polycomb-repressed | Polycomb-repressed | Polycomb-repressed | Polycomb-repressed |
| rs713586 | cg16888658 | Promoter-associated | Promoter-associated | Promoter-associated | Promoter-associated | Promoter-associated | Promoter-associated | Promoter-associated | Enhancer | Promoter-associated | Promoter-associated | Promoter-associated |
| rs713586 | cg23809645 | Quiescent | Promoter-associated | Quiescent | Quiescent | Quiescent | Quiescent | Promoter-associated | Quiescent | Promoter-associated | Promoter-associated | Promoter-associated |
| rs713586 | cg26038461 | Quiescent | Enhancer | Quiescent | Quiescent | Quiescent | Quiescent | Enhancer | Quiescent | Quiescent | Quiescent | Enhancer |
| rs713586 | cg27107076 | Active transcription | Promoter-associated | Promoter-associated | Promoter-associated | Promoter-associated | Promoter-associated | Enhancer | Enhancer | Quiescent | Enhancer | Enhancer |
| rs718314 | cg02058108 | Promoter-associated | Promoter-associated | Promoter-associated | Promoter-associated | Promoter-associated | Promoter-associated | Promoter-associated | Enhancer | Polycomb-repressed | Promoter-associated | Promoter-associated |
| rs7481311 | cg06731443 | Promoter-associated | Promoter-associated | Promoter-associated | Promoter-associated | Promoter-associated | Promoter-associated | Promoter-associated | Promoter-associated | Enhancer | Promoter-associated | Promoter-associated |
| rs7481311 | cg14346046 | Promoter-associated | Promoter-associated | Promoter-associated | Promoter-associated | Promoter-associated | Promoter-associated | Promoter-associated | Promoter-associated | Enhancer | Promoter-associated | Promoter-associated |
| rs7481311 | cg18117895 | Promoter-associated | Promoter-associated | Promoter-associated | Promoter-associated | Promoter-associated | Promoter-associated | Promoter-associated | Promoter-associated | Promoter-associated | Promoter-associated | Promoter-associated |
| rs7498665 | cg00201760 | Polycomb-repressed | Quiescent | Quiescent | Quiescent | Polycomb-repressed | Quiescent | Quiescent | Enhancer | Enhancer | Quiescent | Polycomb-repressed |
| rs7498665 | cg00348858 | Promoter-associated | Promoter-associated | Promoter-associated | Promoter-associated | Promoter-associated | Promoter-associated | Promoter-associated | Promoter-associated | Promoter-associated | Promoter-associated | Promoter-associated |
| rs7498665 | cg00489954 | Active transcription | Active transcription | Active transcription | Active transcription | Active transcription | Active transcription | Active transcription | Active transcription | Active transcription | Active transcription | Active transcription |
| rs7498665 | cg01378222 | Enhancer | Enhancer | Quiescent | Quiescent | Enhancer | Quiescent | Enhancer | Enhancer | Enhancer | Quiescent | Enhancer |
| rs7498665 | cg01621080 | Promoter-associated | Enhancer | Quiescent | Quiescent | Quiescent | Enhancer | Enhancer | Enhancer | Quiescent | Quiescent | Enhancer |
| rs7498665 | cg03300649 | Quiescent | Enhancer | Quiescent | Quiescent | Quiescent | Quiescent | Enhancer | Enhancer | Enhancer | Quiescent | Enhancer |
| rs7498665 | cg04270652 | Polycomb-repressed | Enhancer | Quiescent | Quiescent | Quiescent | Quiescent | Quiescent | Enhancer | Quiescent | Quiescent | Enhancer |
| rs7498665 | cg08180572 | Quiescent | Enhancer | Quiescent | Quiescent | Promoter-associated | Quiescent | Quiescent | Enhancer | Quiescent | Quiescent | Enhancer |
| rs7498665 | cg08761264 | Promoter-associated | Promoter-associated | Promoter-associated | Promoter-associated | Promoter-associated | Promoter-associated | Promoter-associated | Promoter-associated | Promoter-associated | Promoter-associated | Promoter-associated |
| rs7498665 | cg09754948 | Promoter-associated | Promoter-associated | Promoter-associated | Promoter-associated | Promoter-associated | Promoter-associated | Promoter-associated | Promoter-associated | Promoter-associated | Promoter-associated | Promoter-associated |
| rs7498665 | cg26792089 | Polycomb-repressed | Quiescent | Quiescent | Quiescent | Polycomb-repressed | Quiescent | Quiescent | Enhancer | Enhancer | Quiescent | Polycomb-repressed |
| rs7498665 | cg27413008 | Polycomb-repressed | Quiescent | Quiescent | Quiescent | Polycomb-repressed | Quiescent | Quiescent | Enhancer | Enhancer | Quiescent | Polycomb-repressed |
| rs984222 | cg07961512 | Polycomb-repressed | Polycomb-repressed | Polycomb-repressed | Polycomb-repressed | Polycomb-repressed | Polycomb-repressed | Polycomb-repressed | Polycomb-repressed | Polycomb-repressed | Polycomb-repressed | Polycomb-repressed |
| rs984222 | cg17592360 | Promoter-associated | Polycomb-repressed | Polycomb-repressed | Polycomb-repressed | Polycomb-repressed | Polycomb-repressed | Polycomb-repressed | Polycomb-repressed | Promoter-associated | Promoter-associated | Promoter-associated |
