# Supplementary material for: Many obesity-associated SNPs strongly associate with DNA methylation changes at proximal promoters and enhancers
Source: Genome Med. 2015 Oct 8;7:103. doi: 10.1186/s13073-015-0225-4 (PMC4599317; doi:10.1186/s13073-015-0225-4)
Supplement: Additional file 8: — eQTLs found in four eQTL databases for each of the significant 28 SNPs. 1Investigated tissues: subcutaneous adipose tissue, aorta artery, tibial artery, esophagus mucosa, esophagus muscularis, heart left ventricle, lung, skeletal muscle, tibial nerve, sun-exposed skin, lower leg, stomach, thyroid, whole blood. 2Investigated tissues/cell lines: lymphoblastoid cell line (LCL), liver, monocytes, fibroblasts, T cells, brain cortex. 3Investigated tissues/cell lines: lymphoblastoid cell line (LCL), liver, brain cerebellum, brain frontal cortex, brain temporal cortex, brain pons. (DOCX 25 kb) [file 13073_2015_225_MOESM8_ESM.docx]

| **Database** | **GTEx eQTL IGV browser^1^ [45]** | | **eQTL resources from the Gilad/Pritchard group^2^ [82]** | | **eQTL Browser - National Center for Biotechnology Information^3^** | | **Blood eQTL browser [46]** |
| --- | --- | --- | --- | --- | --- | --- | --- |
| SNP | SNP itself | SNPs in strong LD with the SNP (r² > 0.8) | SNP itself | SNPs in strong LD with the SNP (r² > 0.8) | SNP itself | SNPs in strong LD with the SNP (r² > 0.8) | SNP itself |
| rs1011731 |  |  |  |  |  |  | *Z97195.1-1*, *C1orf105*, *PIGC* |
| rs10150332 |  |  |  |  |  |  |  |
| rs1055144 |  |  |  |  |  |  |  |
| rs10767664 |  |  |  |  |  |  |  |
| rs10769908 | *STK33* (tibial artery) | *STK33* (tibial artery, esophagus muscularis, thyroid), *TRIM66* (whole blood, sun-exposed skin) | *EIF3F* (LCLs) | *EIF3F* (LCLs), *UBTFL1* (LCLs) |  |  |  |
| rs10838738 | *C1QTNF4* (subcutaneous adipose, skeletal muscle, sun-exposed skin, whole blood, lung, esophagus mucosa, tibial artery) | *C1QTNF4* (subcutaneous adipose, tibial artery, esophagus mucosa, lung, skeletal muscle,sun-exposed skin, whole blood), *LRP4* (tibial nerve), *MADD* (esophagus muscularis), *PACSIN3* (esophagus mucosa) | *C1QTNF4* (LCLs), *SPI1* (LCLs), *MTCH2* (liver), *MYBPC3* (liver) | *C1QTNF4* (LCLs), *SPI1* (LCLs, monocytes), *MTCH2* (liver, LCLs), *MYBPC3* (liver), *PSMC3* (liver), *MYBPC3* (monocytes, liver), *SPI1*, and *DDB2* (LCLs), *NR1H3* (monocytes), *ARFGAP2* (LCLs) | *CELF1* (liver) | *CELF1* (liver), *SPI1* (LCLs) | *CELF1*, *FNBP4*, *NUP160* |
| rs1152846 |  |  |  |  |  |  |  |
| rs12517906 |  |  |  |  |  |  |  |
| rs1443512 |  |  |  |  |  |  |  |
| rs17782313 |  |  |  | *PIGN* (cortex, monocytes, liver, LCLs), *KIAA1468* (monocytes) |  |  |  |
| rs1878047 |  |  |  |  |  |  | *ETFB* |
| rs1927702 |  | *CCDC171* (esophagus mucosa) |  | *CCDC171* (LCLs) |  |  |  |
| rs206936 |  |  |  | *NUDT3* (monocytes) |  |  |  |
| rs2112347 |  | *POC5* (thyroid) |  | *POC5* (monocytes) |  |  | *POC5* |
| rs2241423 |  | *IQCH-AS1* (tibial artery, aorta artery, esophagus muscularis, whole blood) |  |  |  | *CLN6* (LCLs) | *MAP2K5* |
| rs2287019 |  |  |  |  |  |  | *VASP*, *FBXO46* |
| rs2444217 |  |  |  |  |  |  | *ADCY9* |
| rs2815752 | *RP11-292O17.1* (tibial nerve, thyroid) | *RP11-292O17.1* (tibial nerve, thyroid) |  |  |  |  | *CELF2* |
| rs3934834 |  |  |  | *ADAT3A* (LCLs), *NOC2L* (LCLs) |  |  | *HES4* |
| rs652722 |  |  |  |  |  |  |  |
| rs6784615 |  | *ITIH4-AS1* (aorta artery), *PPM1M* (tibial artery, tibial nerve, skeletal muscle), *NT5DC2* (sun-exposed skin), *GLYCTK* (whole blood) |  | *MANF* (liver), *PPM1M* (liver), *NT5DC2* (LCLs, monocytes), *GLYCTK* (monocytes), *ITIH4* (monocytes), *STAB1* (monocytes) |  |  | *STAB1*, *NT5DC2*, *SPCS1*, *NISCH* |
| rs6795735 |  |  |  |  |  |  |  |
| rs6861681 |  |  | *CPEB4* (LCLs) | *CPEB4* (LCLs, liver, monocytes) | *CPEB4* (brain cerebellum, brain frontal cortex, brain temporal cortex, brain pons) | *CPEB4* (brain cerebellum, brain frontal cortex, brain temporal cortex, brain pons) | *MED30*, *CPEB4* |
| rs713586 | *ADCY3* (whole blood), *RP11-443B20.1* (whole blood) | *ADCY3* (whole blood), *RP11-443B20.1* (whole blood) | *ADCY3* (LCLs, monocytes) | *ADCY3* (LCLs, monocytes) |  |  | *CENPO*, *ADCY3*, *DNAJC27* |
| rs718314 |  |  |  | *KIN* (liver), *SURB7* (liver) |  |  |  |
| rs7481311 |  | *LIN7C* (tibial artery) |  |  |  |  |  |
| rs7498665 | *TUFM* (whole blood, sun-exposed skin, thyroid, tibial artery), *SULT1A1* (thyroid), *SULT1A2* (blood, nerve tibial, subcutaneous adipose, esophagus muscularis), *CDC37P1* (lung, subcutaneous adipose, thyroid, tibial artery, esophagus mucosa, artery aorta, sun-exposed skin), *MIR4721* (blood, tibial artery), *NPIPB7* (esophagus muscularis, thyroid), *RP11-1348G14.4* (tibial artery, lung, nerve tibial), *RP11-435I10.5* (nerve tibial, thyroid), *RP11-1348G14.6* (thyroid), *EIF3C* (thyroid) | *TUFM* (whole blood, sun-exposed skin, thyroid, tibial artery), *SULT1A1* (thyroid), *SULT1A2* (blood, nerve tibial, subcutaneous adipose, esophagus muscularis), *CDC37P1* (lung, subcutaneous adipose, thyroid, tibial artery, esophagus mucosa, artery aorta, sun-exposed skin), *MIR4721* (blood, tibial artery), *NPIPB7* (esophagus muscularis, thyroid), *RP11-1348G14.4* (tibial artery, lung, nerve tibial), *RP11-435I10.5* (nerve tibial, thyroid), *RP11-1348G14.6* (thyroid), *EIF3C* (thyroid) | *EIF3C* (LCLs), *SPNS1* (LCLs) | *EIF3C* (liver, LCLs), *TUFM* (LCLs), *CLN3* (LCLs), *CDIPT* (LCLs), *SULT1A1* (monocytes), *SULT1A4* (monocytes), *SPNS1* (LCLs), *LOC112869* (monocytes), *SPIN1* (monocytes) |  | *CCDC120* (liver), *EIF3C* (liver) | *STAT1*, *TUFM*, *SPNS1* |
| rs984222 |  | *WARS2* (tibial artery, skeletal muscle, sun-exposed skin, stomach), *RP11418J17.1* (esophagus muscularis, lung) | *MB21D2* (LCLs) | *WARS2* (LCLs) |  |  | *WARS2* |
