# Supplementary material for: Many obesity-associated SNPs strongly associate with DNA methylation changes at proximal promoters and enhancers
Source: Genome Med. 2015 Oct 8;7:103. doi: 10.1186/s13073-015-0225-4 (PMC4599317; doi:10.1186/s13073-015-0225-4)
Supplement: Additional file 10: — Replication of the 107 SNP-CpG associations found in blood. *Coefficient of the linear model associated with the obesity-associated SNP: positive for increased methylation with presence of risk allele; coefficients are calculated using M values. Coefficients with a hash symbol correspond to associations with raw p value < 0.05 and coefficients in bold correspond to associations with q value < 0.05. (DOCX 31 kb) [file 13073_2015_225_MOESM10_ESM.docx]

| SNP | CpG | Coefficient in blood (n=355)* | Coefficient in skin fibroblasts (n=62)* | Coefficient in cerebellum (n=119)* | Coefficient in frontal cortex (n=133)* | Coefficient in caudal pons (n=125)* | Coefficient in temporal cortex (n=127)* | Coefficient in subcutaneous adipose tissue (n=149)* | Coefficient in visceral adipose tissue (n=149)* |
| --- | --- | --- | --- | --- | --- | --- | --- | --- | --- |
| rs1011731 | cg13446689 | 0.357 | **0.254^#^** |  |  |  |  | 0.0016 | -0.0788 |
| rs10150332 | cg07177395 | -0.138 |  |  |  |  |  |  |  |
| rs1055144 | cg00935653 | 0.116 | 0.435 |  |  |  |  |  |  |
| rs1055144 | cg03190219 | 0.307 | 0.496 |  |  |  |  |  |  |
| rs1055144 | cg05149343 | 0.111 | 0.384 |  |  |  |  |  |  |
| rs1055144 | cg09596116 | 0.19 | 0.0786 |  |  |  |  |  |  |
| rs1055144 | cg13710556 | 0.214 | -0.0152 |  |  |  |  |  |  |
| rs1055144 | cg15575538 | 0.234 | -0.257 |  |  |  |  |  |  |
| rs10767664 | cg09781307 | -0.306 |  |  |  |  |  |  |  |
| rs10767664 | cg10635145 | 0.322 |  |  |  |  |  |  |  |
| rs10767664 | cg18117895 | -0.0921 |  |  |  |  |  |  |  |
| rs10767664 | cg26949694 | 0.137 |  |  |  |  |  |  |  |
| rs10769908 | cg01677628 | -0.285 |  |  |  |  |  |  |  |
| rs10769908 | cg07138994 | -0.261 |  |  |  |  |  |  |  |
| rs10769908 | cg10639395 | -0.182 |  |  |  |  |  |  |  |
| rs10769908 | cg27431761 | -0.354 |  |  |  |  |  |  |  |
| rs10838738 | cg00214780 | 0.1 | 0.00616 |  |  |  |  |  |  |
| rs10838738 | cg04959790 | -0.118 | 0.469**^#^** |  |  |  |  |  |  |
| rs10838738 | cg05377527 | 0.126 | -0.284 |  |  |  |  |  |  |
| rs10838738 | cg05585544 | -0.171 | **-0.638^#^** | -0.0741 | -0.0460 | -0.0310 | -0.0640 |  |  |
| rs10838738 | cg13308137 | -0.629 | -0.104 |  |  |  |  |  |  |
| rs10838738 | cg14232165 | 0.17 | 0.0924 |  |  |  |  |  |  |
| rs10838738 | cg18512352 | -0.249 | **-0.467^#^** |  |  |  |  |  |  |
| rs10838738 | cg20135002 | -0.252 | -0.0626 |  |  |  |  |  |  |
| rs1152846 | cg04441577 | 0.31 | -0.0822 |  |  |  |  |  |  |
| rs12517906 | cg00514575 | 1.18 | -0.716 |  |  |  |  |  |  |
| rs12517906 | cg13004587 | 0.213 | -0.000381 |  |  |  |  |  |  |
| rs1443512 | cg07731191 | 0.342 | 0.425**^#^** |  |  |  |  |  |  |
| rs1443512 | cg16295056 | 0.304 | -0.0573 |  |  |  |  |  |  |
| rs1443512 | cg16305379 | -0.138 | 0.0429 |  |  |  |  |  |  |
| rs1443512 | cg25024717 | 0.145 | 0.729**^#^** |  |  |  |  |  |  |
| rs17782313 | cg22549408 | -0.159 |  |  |  |  |  |  |  |
| rs1878047 | cg04588972 | -0.0582 | 0.0388 |  |  |  |  |  |  |
| rs1878047 | cg14884932 | -0.278 | **-0.973^#^** |  |  |  |  |  |  |
| rs1878047 | cg15497724 | -0.337 | -0.54**^#^** |  |  |  |  |  |  |
| rs1927702 | cg01560422 | 0.156 | 0.37 |  |  |  |  |  |  |
| rs206936 | cg23117447 | 0.0909 | 0.0491 |  |  |  |  |  |  |
| rs2112347 | cg03649429 | 0.551 |  |  |  |  |  |  |  |
| rs2241423 | cg07010088 | -0.158 | 0.116 |  |  |  |  |  |  |
| rs2241423 | cg09469610 | -0.119 | 0.223 |  |  |  |  |  |  |
| rs2241423 | cg09917562 | 0.429 | 0.154 |  |  |  |  |  |  |
| rs2241423 | cg24579218 | 0.695 | 0.398 |  |  |  |  |  |  |
| rs2241423 | cg26545918 | -0.135 | -0.0298 |  |  |  |  |  |  |
| rs2241423 | cg27219399 | -0.233 | -0.459 |  |  |  |  |  |  |
| rs2287019 | cg02473103 | 0.0619 | 0.0635 |  |  |  |  |  |  |
| rs2287019 | cg04282912 | -0.482 | -0.0675 |  |  |  |  |  |  |
| rs2287019 | cg13320842 | -0.38 | -0.422 |  |  |  |  |  |  |
| rs2287019 | cg18735402 | 0.499 | -0.581 |  |  |  |  |  |  |
| rs2287019 | cg19822309 | 0.415 | -0.202 |  |  |  |  |  |  |
| rs2287019 | cg20434926 | 0.304 | -0.19 |  |  |  |  |  |  |
| rs2444217 | cg00834536 | 0.105 | -0.0789 |  |  |  |  |  |  |
| rs2444217 | cg07628416 | -0.223 | 0.233 |  |  |  |  |  |  |
| rs2444217 | cg08098950 | -0.201 | -0.0495 |  |  |  |  |  |  |
| rs2444217 | cg09300795 | 0.218 | **-0.652^#^** |  |  |  |  |  |  |
| rs2815752 | cg09256413 | -0.168 | 0.0763 |  |  |  |  |  |  |
| rs3934834 | cg00305285 | 0.0922 | 0.0804 |  |  |  |  |  |  |
| rs3934834 | cg02105666 | -0.181 | -1.03**^#^** |  |  |  |  |  |  |
| rs3934834 | cg02341264 | -0.241 | -0.993**^#^** |  |  |  |  |  |  |
| rs3934834 | cg07549208 | -0.285 | -0.691 |  |  |  |  |  |  |
| rs3934834 | cg07787977 | 0.091 | -0.000887 |  |  |  |  |  |  |
| rs3934834 | cg09363892 | -0.176 | -0.106 |  |  |  |  |  |  |
| rs3934834 | cg11200797 | -0.682 | -0.247 |  |  |  |  |  |  |
| rs3934834 | cg15500259 | 0.156 | -0.189 |  |  |  |  |  |  |
| rs3934834 | cg15576492 | -1.53 | -0.127 |  |  |  |  |  |  |
| rs3934834 | cg17021880 | 0.576 | 0.484 |  |  |  |  |  |  |
| rs3934834 | cg18432292 | -0.145 | -0.355 |  |  |  |  |  |  |
| rs3934834 | cg20685419 | -0.0848 | -0.134 |  |  |  |  |  |  |
| rs3934834 | cg21139076 | -0.262 | -0.247 |  |  |  |  |  |  |
| rs3934834 | cg22044028 | -1.28 | -0.113**^#^** |  |  |  |  |  |  |
| rs3934834 | cg22864340 | -0.263 | 0.0503 |  |  |  |  |  |  |
| rs652722 | cg11385473 | 0.201 | 0.00571 | 0.131 | -0.0371 | 0.0200 | -0.0446 |  |  |
| rs6784615 | cg04865290 | 0.996 |  |  |  |  |  |  |  |
| rs6784615 | cg07615364 | 0.208 |  |  |  |  |  |  |  |
| rs6784615 | cg11645453 | 0.498 |  |  |  |  |  |  |  |
| rs6784615 | cg16362603 | 0.315 |  |  |  |  |  |  |  |
| rs6784615 | cg18404041 | -0.247 |  |  |  |  |  |  |  |
| rs6795735 | cg23078228 | -0.16 | 0.104 |  |  |  |  |  |  |
| rs6861681 | cg06889108 | -0.117 |  |  |  |  |  |  |  |
| rs6861681 | cg18693985 | -1.13 |  |  |  |  |  |  |  |
| rs6861681 | cg18757087 | -0.156 |  |  |  |  |  |  |  |
| rs6861681 | cg21566177 | -0.53 |  |  |  |  |  |  |  |
| rs713586 | cg01884057 | 0.752 |  |  |  |  |  |  |  |
| rs713586 | cg09505516 | -0.38 |  |  |  |  |  |  |  |
| rs713586 | cg11023668 | -0.636 |  |  |  |  |  |  |  |
| rs713586 | cg16302441 | -0.147 |  |  |  |  |  |  |  |
| rs713586 | cg16888658 | -0.166 |  |  |  |  |  |  |  |
| rs713586 | cg23809645 | 0.208 |  |  |  |  |  |  |  |
| rs713586 | cg26038461 | -0.111 |  |  |  |  |  |  |  |
| rs713586 | cg27107076 | -0.174 |  |  |  |  |  |  |  |
| rs718314 | cg02058108 | 0.174 | 0.148 |  |  |  |  |  |  |
| rs7481311 | cg06731443 | 0.262 | 0.138 |  |  |  |  |  |  |
| rs7481311 | cg14346046 | 0.215 | **-0.553^#^** |  |  |  |  |  |  |
| rs7481311 | cg18117895 | -0.0537 | **0.175^#^** |  |  |  |  |  |  |
| rs7498665 | cg00201760 | -0.0697 |  |  |  |  |  |  |  |
| rs7498665 | cg00348858 | -0.155 |  |  |  |  |  |  |  |
| rs7498665 | cg00489954 | 0.138 |  |  |  |  |  |  |  |
| rs7498665 | cg01378222 | 0.182 |  |  |  |  |  |  |  |
| rs7498665 | cg01621080 | -0.17 |  |  |  |  |  |  |  |
| rs7498665 | cg03300649 | -0.0941 |  |  |  |  |  |  |  |
| rs7498665 | cg04270652 | -0.367 |  |  |  |  |  |  |  |
| rs7498665 | cg08180572 | -0.146 |  |  |  |  |  |  |  |
| rs7498665 | cg08761264 | -0.121 |  |  |  |  |  |  |  |
| rs7498665 | cg09754948 | 0.238 |  |  |  |  |  |  |  |
| rs7498665 | cg26792089 | -0.0819 |  |  |  |  |  |  |  |
| rs7498665 | cg27413008 | -0.363 |  |  |  |  |  |  |  |
| rs984222 | cg07961512 | 0.378 |  |  |  |  |  |  |  |
| rs984222 | cg17592360 | 0.153 |  |  |  |  |  |  |  |
